# A systematic review of epidemiological modelling in response to lumpy skin disease outbreaks

**DOI:** 10.3389/fvets.2024.1459293

**Published:** 2024-09-23

**Authors:** Simin Lee, Christopher M. Baker, Emily Sellens, Mark A. Stevenson, Sharon Roche, Robyn N. Hall, Andrew C. Breed, Simon M. Firestone

**Affiliations:** ^1^Melbourne Veterinary School, Faculty of Science, The University of Melbourne, Parkville, VIC, Australia; ^2^School of Mathematics and Statistics, Faculty of Science, The University of Melbourne, Parkville, VIC, Australia; ^3^Melbourne Centre for Data Science, The University of Melbourne, Parkville, VIC, Australia; ^4^The Centre of Excellence for Biosecurity Risk Analysis, School of Biosciences, The University of Melbourne, Parkville, VIC, Australia; ^5^Epidemiology, Surveillance and Laboratory Section, Australian Government Department of Agriculture, Fisheries and Forestry, Canberra, ACT, Australia; ^6^Ausvet Pty Ltd., Canberra, ACT, Australia

**Keywords:** lumpy skin disease, outbreak response, modelling workflow, decision making, systematic review

## Abstract

Lumpy skin disease (LSD) is an infectious disease currently spreading worldwide and poses a serious global threat. However, there is limited evidence and understanding to support the use of models to inform decision-making in LSD outbreak responses. This review aimed to identify modelling approaches that can be used before and during an outbreak of LSD, examining their characteristics and priorities, and proposing a structured workflow. We conducted a systematic review and identified 60 relevant publications on LSD outbreak modelling. The review identified six categories of question to be addressed following outbreak detection (origin, entry pathway, outbreak severity, risk factors, spread, and effectiveness of control measures), and five analytical techniques used to address them (descriptive epidemiology, risk factor analysis, spatiotemporal analysis, dynamic transmission modelling, and simulation modelling). We evaluated the questions each analytical technique can address, along with their data requirements and limitations, and accordingly assigned priorities to the modelling. Based on this, we propose a structured workflow for modelling during an LSD outbreak. Additionally, we emphasise the importance of pre-outbreak preparation and continuous updating of modelling post-outbreak for effective decision-making. This study also discusses the inherent limitations and uncertainties in the identified modelling approaches. To support this workflow, high-quality data must be collected in standardised formats, and efforts should be made to reduce inherent uncertainties of the models. The suggested modelling workflow can be used as a process to support rapid response for countries facing their first LSD occurrence and can be adapted to other transboundary diseases.

## 1 Introduction

Transboundary animal diseases are infectious diseases that cross borders and affect animal and human populations and ecosystems. Diseases such as lumpy skin disease (LSD), foot-and-mouth disease (FMD), African swine fever (ASF), and high pathogenicity avian influenza (HPAI) represent serious threats to humanity as they spread to neighbouring countries through trade in live animals, animal products, and vectors, causing major animal health and economic crises (1, 2). The spread of these diseases has caused significant disruptions to global food systems and has highlighted the need for effective surveillance and control measures to reduce the spread and impact of transboundary animal diseases.

Rapid development and implementation of response strategies after the first occurrence of a transboundary animal disease in a country are essential for mitigating impacts, with the goal of containing the situation and hopefully achieving elimination locally. In 2001, when FMD was confirmed in Essex in the UK, the government swiftly implemented control strategies, including a ban on livestock movement, epidemiological investigation, culling within 24 h of detection, and a prohibition on all exports of meat and live animals, which helped alleviate the risk of international spread and minimise losses (3, 4). The COVID19 pandemic has highlighted the urgent need for prompt response in infectious disease outbreaks. Countries are systematically preparing responses for future animal disease emergencies based on experience and available scientific information (5, 6). However, there is a lack of useful categorisation regarding the modelling necessary to understand and respond to the complex situations that arise during an outbreak.

In the context of disease outbreaks, mathematical and statistical models can estimate the spread and impact of a disease and evaluate the potential effects of control measures to inform when and where they should be implemented (7–9). In addition, models can be used for timely monitoring of the effects of interventions, identifying potential gaps between the available and required resources to support control measures, and determining when and where strategic or tactical changes may be needed (10). The information provided supports decision-making and helps respond more quickly and effectively. Therefore, modelling is an essential tool for mitigating the spread of infectious diseases and preventing their escalation into animal health emergencies.

LSD is a viral disease primarily affecting cattle that can spread by various transmission pathways. The disease is mainly reported to be mechanically transmitted from clinically infected or subclinically infected cattle by biting arthropods (11). Direct contact with infected animals, or via contaminated objects may play a smaller and less defined role in transmission (12). Additionally, indirect transmission between cattle through shared water troughs has been reported (13). However, the extent to which each of these transmission routes contributes to the spread of the disease is not well-established. LSD is characterised by nodular lesions in the skin and other epithelial tissues (e.g., gastrointestinal tract, respiratory tract), fever, loss of appetite, and decreased milk production, resulting in significant production losses (12, 14). The virus is infectious and spreads quickly within a country after the first occurrence (15). To effectively respond to this, a timely modelling approach is necessary. However, each modelling approach has varying resource requirements and considerations, and the resulting uncertainty complicates decision-making. Therefore, understanding of the timeliness of modelling approaches is an important aspect in planning and executing response measures appropriately.

We conducted a systematic review of eligible studies to identify and assess currently available models for LSD and their timeliness for application, and to review models or analyses that can be developed in advance of an outbreak. Therefore, in this study, we examine what modelling can be undertaken before and after detection of the first LSD occurrence in a country, develop a workflow for performing such modelling, and evaluate the limitations of models or the workflow. We defined modelling as encompassing all analytical approaches that can describe (i.e., back- and now-cast) and forecast the progress of epidemics, including analysing epidemic curves, spatiotemporal trends and risk factors, and quantitative approaches that use data to guide inference and decision-making. This definition is therefore not limited to simulation modelling.

## 2 Materials and methods

### 2.1 Study protocol and eligibility criteria

This review procedure followed the Preferred Reporting Items for Systematic Review and Meta-Analysis Protocols (PRISMA-P) guidelines (16, 17). Table 1 summarises the eligibility criteria and search strings used. Based on initial review of the literature and consultations with government veterinarians and further experts and stakeholders included as co-authors of this paper, we categorised the six questions of interest to scientists and decision-makers in an LSD outbreak: origin, entry pathway, outbreak severity, risk factors, spread, and effectiveness of control measures. We included only studies using suitable analytical methods to answer questions under these categories. The target population and disease were cattle and LSD, respectively. The review excluded research that examined capripoxviruses other than LSD virus (LSDV). Since we included various research methodologies in the review, it was not possible to establish a comprehensive intervention and comparator. Therefore, we assigned the corresponding criteria only to risk factor analyses with distinct interventions and comparators. There were no restrictions on publication year or language.

**Table 1:** Eligibility criteria and search strings.

	Inclusion criteria	Exclusion criteria
Study type	Observational studies	Experimental studies (including clinical trials or randomized controlled trials)
Population	Cattle	Other animals except cattle
Intervention	Lumpy skin disease virus Potential risks^a^	Other capripoxviruses
Comparator	Population not exposed to potential risks^a^	
Outcomes	Outcomes to get answers to the following key questions. Where did the disease originate? What is the introduction pathway to the country? How severe is the outbreak? What are the risk factors for the spread between farms? What will the disease spread be like after the outbreak? Which control measures are effective and efficient in controlling the outbreak?	Others

a Depends on the study type as some do not have intervention and comparator.

Search strings (Lumpy Skin disease OR LSD) AND (modeling OR modelling) AND (epidemiology OR outbreak).

### 2.2 Information sources and study selection

We searched the databases (PubMed, Web of Science, Scopus, and Google scholar) on 8 Feb 2023 using the search string “(Lumpy Skin disease OR LSD) AND (modeling OR modelling) AND (epidemiology OR outbreak)”. When additional publications related to the review’s objectives were discovered through further searches, these were also included in the study selection process. We uploaded search results to Zotero 6.0 (Corporation for Digital Scholarship, Virginia, United States) and removed duplicates. Two independent researchers evaluated the eligibility of each study. During the initial assessment, two independent researchers reviewed the titles and abstracts, while the full text was reviewed in the last assessment. Discrepancies were resolved through consensus or independent reviewer mediation.

### 2.3 Data extraction

Two independent researchers extracted the following data from eligible studies: author, publication year, analysis type, sub-categories of analysis type, category of questions of interest, required data, country, and study period. We collected only the data presented in the article; no contact was made with the authors to gather or confirm any information. There were no data assumptions made, and missing data were noted as “not available.”

### 2.4 Summary of review

**Figure 1:**
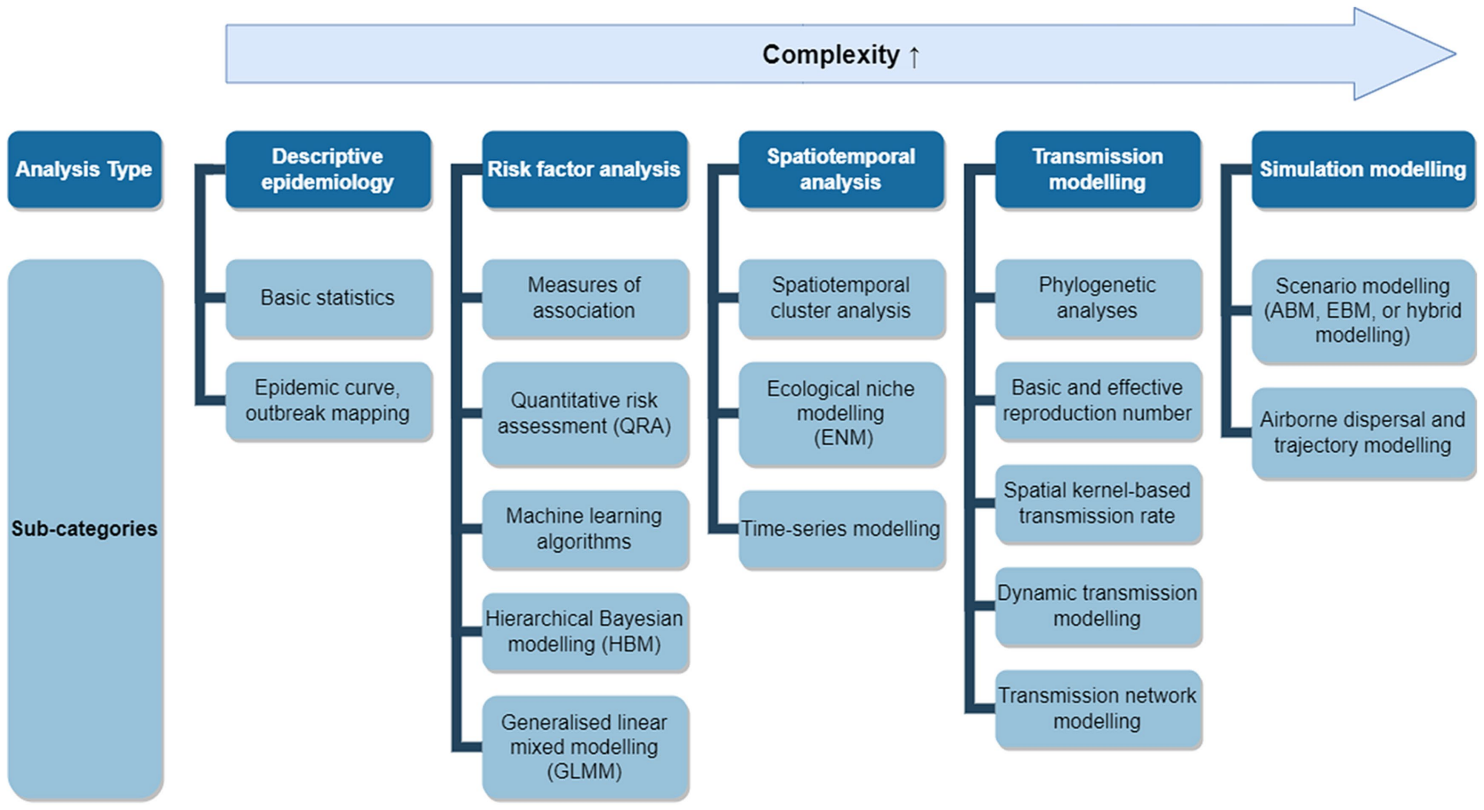
Applicable modelling approaches for an LSD outbreak. Applicable models were grouped into five analysis types with 17 sub-categories. Analysis types positioned further to the right represent more complex modelling approaches and may require additional data, assumptions, and implementation time.

We classified the models used in eligible studies into five over-arching types based on their purposes and further subdivided them into 17 sub-categories based on their method (see Figure 1).

1. Descriptive epidemiology: used to evaluate the severity of LSD outbreaks and spatiotemporal distribution.
  - Basic statistics.
  - Epidemic curve plotting, outbreak mapping, analyses and interpretation.
2. Risk factor analysis: various analytical methods to identify factors contributing to LSD outbreaks.
  - Measures of association.
  - Quantitative risk assessment (QRA).
  - Machine learning algorithms.
  - Hierarchical Bayesian modelling (HBM).
  - Generalised linear mixed modelling (GLMM).
3. Spatiotemporal analysis: a more detailed approach that goes beyond descriptive epidemiology and investigates the timing and location of disease occurrence.
  - Spatiotemporal cluster analysis.
  - Ecological niche modelling (ENM). ENMs identify areas highly suitable for the presence of LSDV or its carrying vectors based on environmental factors, in contrast to Species Distributions Models (SDMs) which estimate the actual distributions of species (18). Therefore, this paper focusing on disease incursion has directed its emphasis solely towards ENMs, not considering SDMs.
  - Time-series modelling.
4. Transmission modelling: explicitly exploring and modelling the underlying mechanisms of how diseases are introduced and spread.
  - Phylogenetic analyses.
  - Basic reproduction number (*R_0_*) and effective reproduction number (*R_E_*) estimation.
  - Spatial kernel-based transmission rate estimation.
  - Dynamic transmission modelling. Dynamic transmission modelling estimates changes in compartments that represent susceptible, infectious, and recovered premises or individuals using fundamental epidemiological parameters (19). When the SIR model incorporates specific intervention strategy scenarios, it is classified as scenario modelling. Conversely, when the SIR model is applied using only the basic epidemiological parameters without integrating specific intervention scenarios, it is categorised as dynamic transmission modelling.
  - Integrated genomic and epidemiological transmission network modelling.
5. Simulation modelling: simulating future transmission patterns and control measure effectiveness.
  - Scenario modelling (agent-based modelling (ABM), equation-based modelling (EBM), or hybrid modelling). Scenario modelling is a strategic planning technique that involves creating and analysing various hypothetical situations or scenarios to understand their potential outcomes and impacts. EBM calculates compartment-specific population changes based on mathematical modelling (20). While ABM simulates a system composed of simplified behaviours of diverse individuals (20). There also exists a hybrid model that combines ABM and EBM.
  - Airborne dispersal and trajectory modelling.

We did not assess the risk of bias across the studies included in our review because our review primarily aimed to provide a descriptive overview of the modelling approaches and their applications to LSD. We graphically represented the timeliness of modelling and the six question categories and assessed each model to determine its potential in addressing each of the six question categories. We listed the analysis type, sub-category of analysis type, what questions the modelling outputs can answer, required data for analysis, priority (low, moderate, and high), and other limitations to assist researchers in selecting the most suitable model for their needs at different stages following an LSD incursion. Priority is context dependent and relates to the questions a model is being used to answer. We assessed model priorities subjectively based on the following four criteria:

1. Urgency: of the questions the modelling outputs are being used to answer,
2. Timeliness: whether the modelling modality can answer those questions in time to be useful for decision support (depending on data and resources),
3. Accuracy: acceptable level of uncertainty given limited resources, and
4. Versatility: the ability of the model to address a variety of questions depending on the changing outbreak situation.

### 2.5 Data analysis and visualisation

We visualised the frequency of modelling approaches (analysis types and sub-categories) utilised in eligible publications using a nested pie chart created in Excel (Microsoft, Redmond, United States). We mapped the year of LSD’s first detection by country using QGIS (version 3.28), based on relevant references (21–32). Additionally, we illustrated the recent spread of LSD in South and Southeast Asia on the map with arrows, based on molecular analysis (33–40).

## 3 Results

### 3.1 Study selection

**Figure 2:**
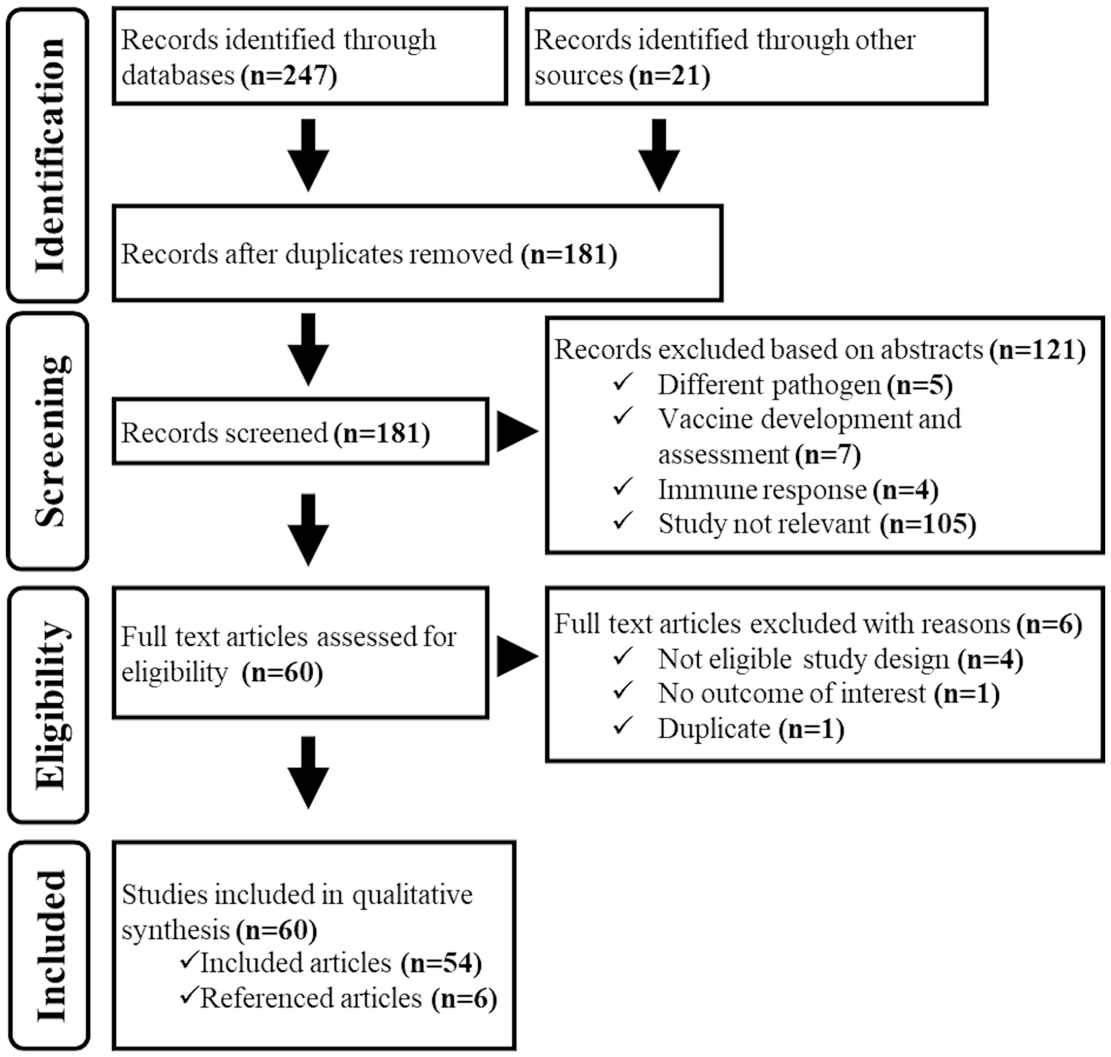
PRISMA-P flowchart representing the screening process for eligible studies.

Database and grey literature searches identified 247 documents matching the searching strings, and we additionally identified 21 documents among those shared by the authors. A total of 181 electronic records remained after removing duplicates. During the title/abstract screening process, we eliminated 121 documents as they did not fit the eligibility criteria. In the full-text screening process, we excluded six more documents due to ineligible study design, outcomes, and duplicate content. As a result, 54 documents were considered suitable for the study. However, we found six additional relevant records by examining the reference lists of these documents. Thus, 60 documents in total were included in the study (Figure 2).

### 3.2 Study characteristics

**Figure 3:**
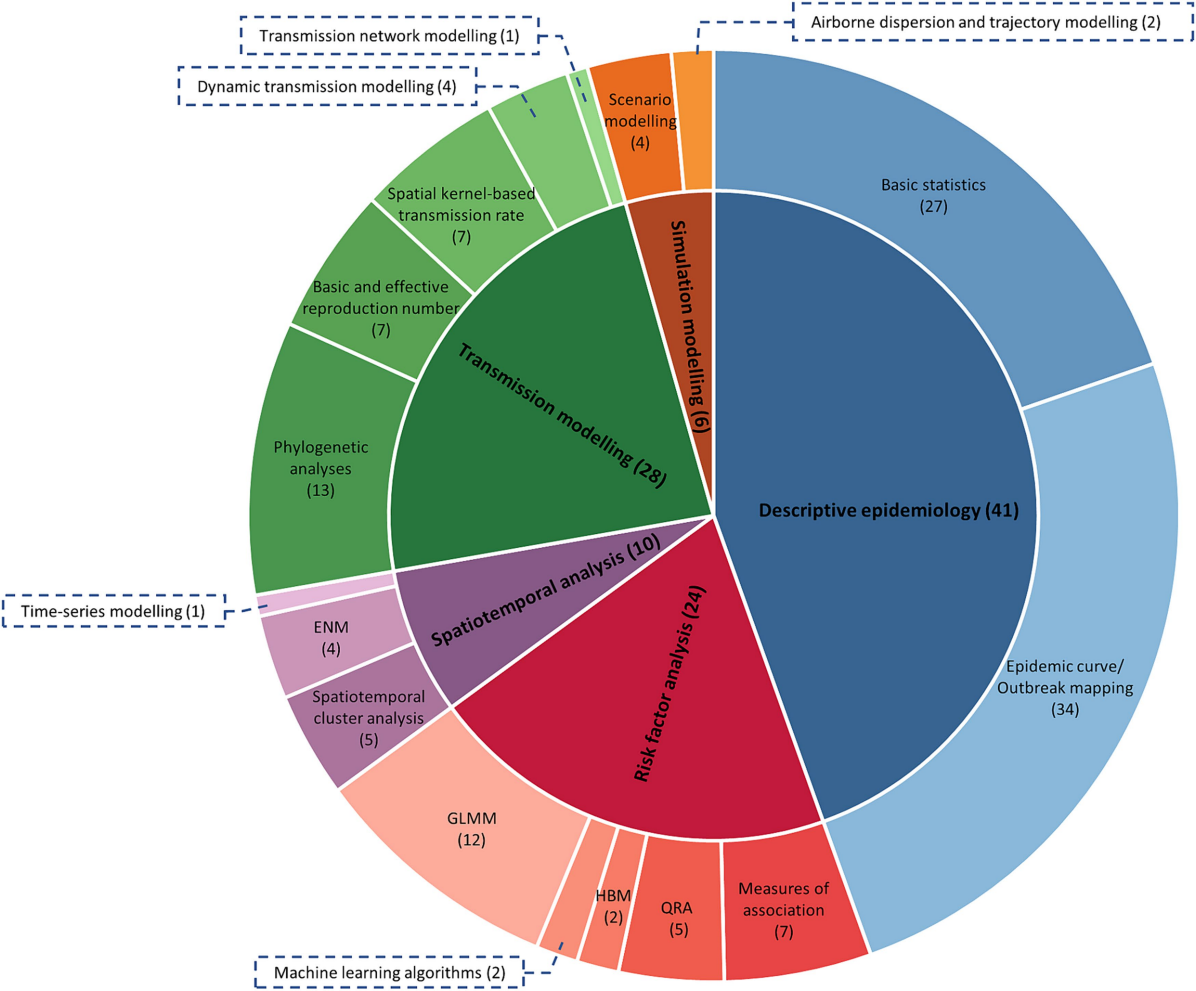
The frequency of each analysis type and sub-category of analysis used in the eligible studies (*n* = 51). Numbers in parentheses indicate the number of papers that used each analysis type or sub-category, noting some studies used more than one type. QRA, quantitative risk assessment; HBM, hierarchical Bayesian modelling; GLMM, generalised linear mixed modelling; ENM, ecological niche modelling.

Supplementary Table S1 provides an overview of features of the 60 studies included in this research (7–9, 13, 15, 30, 33–39, 41–87). The analysis types and sub-categories of models are further illustrated in Figure 3. It is important to note that studies that reported multiple analysis types and sub-categories were included in all relevant sub-categories, resulting in a total count in Figure 3 greater than 60. The most frequent analysis type was descriptive epidemiology, employed in 41 studies, while the least used was simulation modelling, employed in only six studies. Risk factor analysis was reported in 24 studies, spatiotemporal analysis in 10 studies, and transmission modelling in 28 studies. The complexity of data and parameters required to build the models increased in the order of descriptive epidemiology, risk factor analysis, spatiotemporal analysis, transmission modelling, and simulation modelling. As the complexity of these models increased, the number of published studies in each model category decreased. The 60 studies were conducted in different geographical regions, including Africa, the Middle East, Europe, Asia, and Oceania.

### 3.3 Results of individual studies

Out of 41 descriptive epidemiology studies (7–9, 13, 15, 30, 33–35, 37–39, 41–47, 49, 51–54, 58, 61, 63, 65, 68–74, 76, 80, 81, 83, 84, 86), 27 utilised basic descriptive statistics to calculate morbidity and mortality rates for determining outbreak severity. Most studies reported LSD morbidity rates between 5 and 40%, and mortality rates between 0 and 5%. The case fatality rate (CFR) was reported to be <1% in Uganda and Bangladesh, but CFR in Thailand was 10% (38, 74, 86). Given the very low mortality rates in Uganda and Bangladesh and the higher mortality rate in Thailand, the CFR should be interpreted cautiously depending on the region and context (Uganda: 0.03% mortality, 0.72% CFR (74), Bangladesh: 0.26% mortality, 0.97% CFR (86), Thailand: 3.47% mortality, 10.05% CFR (38)). The temporal and spatial patterns of LSD outbreaks using epidemic curves and outbreak mapping were visualised in 34 out of 41 descriptive epidemiology studies. Most studies reported that LSD outbreaks occurred between summer and autumn (fall), except for in Uganda, where a study reported occurrence during the dry season (Dec to Feb) (74). This appears to be due to different periods of increased vector abundance based on the climatic conditions of each country. The abundance of vectors tends to increase during the rainy summer (temperate climate) or wet season (tropical climate), and a pattern is predominantly observed where vector-borne disease occurrence rises in the subsequent season (88, 89). Studies with less than 6 months of observation period were unable to confirm seasonality but did observe a rapid increase in LSD cases between 10 to 20 days after the confirmation of the first case in the country (15, 70). When examining the spatial distribution of LSD outbreaks, it was found that they tended to occur in areas near lakes and rivers (65, 74, 83).

**Figure 4:**
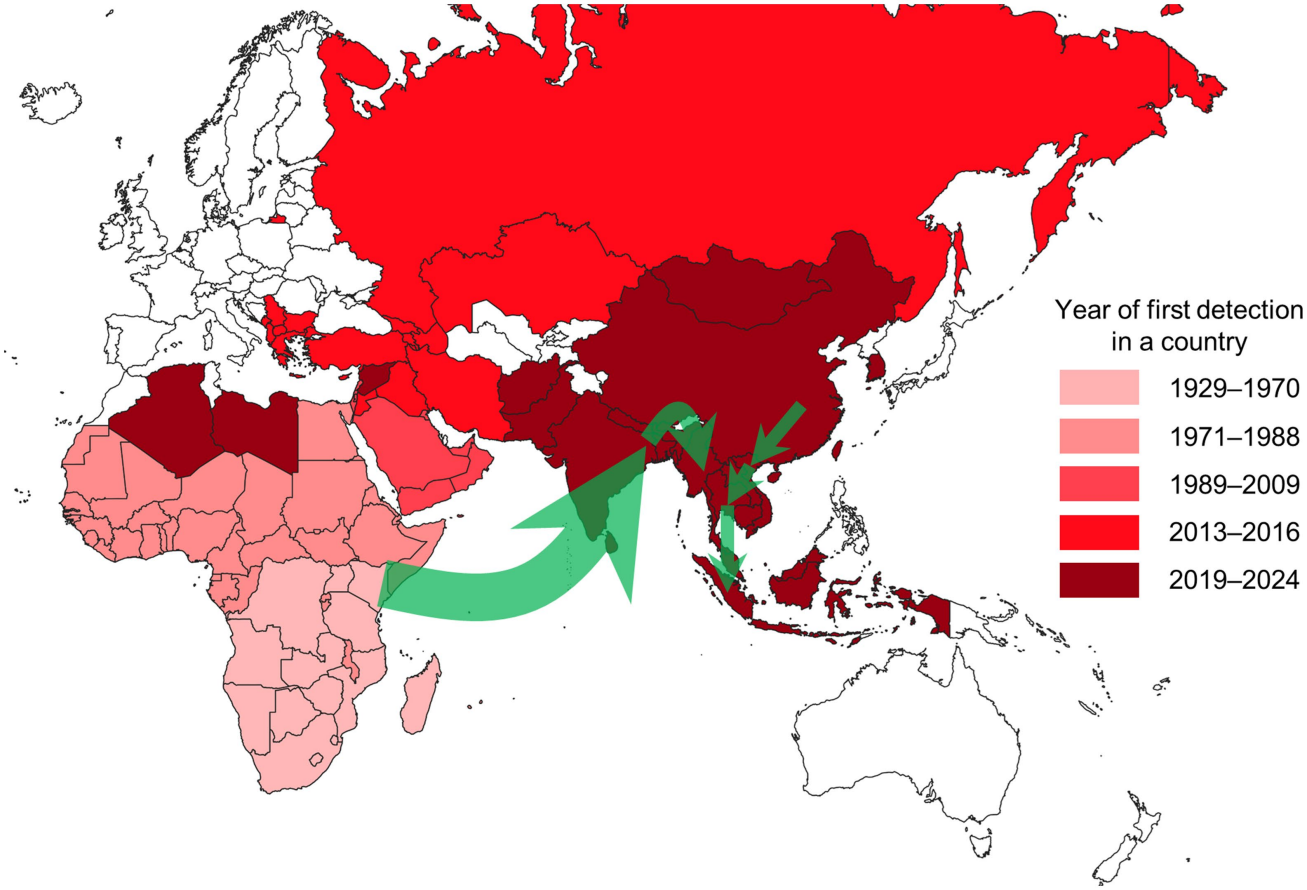
Map showing the year of first LSD detection by country as of August 2024. Countries shaded in white have not reported any LSD outbreaks. The green arrows represent the estimated spread routes of LSD in South and Southeast Asia over the past 5 years. The Americas, where LSD has not been reported, are excluded from the map.

The intercontinental spread of LSD was observed to have originated from endemic regions in Africa and spread to the Middle East during the 1990s and 2000s, reaching Türkiye in 2013, and eventually extending to the Balkans and Eurasia in 2015 and 2016 (Figure 4) (8, 9, 13, 45, 52, 53). In 2019, LSD outbreaks were detected in India (33), China (68), and Bangladesh (34), spreading to Nepal (35), Pakistan (29), Bhutan (29), and Sri Lanka (29). In 2020, LSD spread from China to Vietnam (36), and between 2020 and 2021, the disease further spread to Southeast Asian countries, including Thailand (37), Cambodia (29), Laos (29), Myanmar (39), and Malaysia (29). In 2022, the disease reached Indonesia (83), and in 2023, it appeared in South Korea (29). This demonstrates that LSD has recently spread extensively across countries over a notably brief period.

The spread of LSD into Europe was halted by mass vaccination and stamping out policies in the Balkans. Southeast Asia experienced a rapid spread of LSD among susceptible cattle due to the lack of vaccination before the first outbreak (90). Efforts to achieve control and eradication are being continuously pursued through the prompt implementation and ongoing enforcement of policies such as vaccination, stamping out, movement restrictions, active surveillance, vector control, and financial compensation (37, 91). As such, descriptive epidemiology can aid in understanding the spatiotemporal transmission of the disease and serve as a tool to assess the outbreak and severity of the situation. This can support the interventions needed for the control and prevention of LSD. However, the local spread and daily epidemic curves provided in the studies could not be generalised due to reporting inconsistencies, highlighting the need for additional information and empirical evidence to overcome this limitation.

A total of 24 studies evaluated risk factors for LSD occurrence. As methods for evaluating risk factors, measures of assessment, quantitative risk assessment (QRA), hierarchical Bayesian modelling (HBM), machine learning algorithms, generalised linear mixed modelling (GLMM) were used in seven (41, 43, 59, 63, 73, 83, 86), five (57, 62, 77, 78, 85), two (45, 69), two (42, 82), and 12 papers (9, 48, 59, 63, 65, 66, 72, 73, 75, 80, 83, 86), respectively. Of the 12 studies that utilised GLMMs, nine were suitable for analysing risk factors associated with LSD outbreaks as they assigned the number of LSD outbreaks during the study extents as the dependent variable in GLMMs (48, 59, 63, 66, 73, 75, 80, 83, 86). On the other hand, the remaining three studies employed GLMMs to analyse variables affecting vaccine immunity (9) and vector abundance (65), as well as to estimate parameters used in subsequent modelling processes (72). Therefore, our focus regarding the discussion of risk factors was primarily on the former nine studies. Since HBM was used to identify meteorological risk factors to be included in ENM (45, 69), these risk factors are quite different from those considered in measures of assessment and GLMM. Therefore, the risk factors considered in measures of assessment and GLMM were reviewed separately from those considered in HBM.

Studies using measures of assessment and GLMM commonly identified location (73, 80) and cattle introduction (58, 63, 66, 83) as high-risk factors. This suggests that the risk of LSD occurrence may be higher in areas near rivers or where cattle are introduced from external sources. Inconsistent results were found for other risk factors including age (41, 73, 75, 80), sex (41, 63, 73, 75), breed (41, 63, 66, 73, 75, 80), cattle type (43, 63, 80), altitude (41, 58, 59, 73), communal water trough (58, 63, 66, 75), contact with other animals (73, 75, 86), grazing (58, 66, 75, 83), and herd size (43, 66, 75, 80, 83, 86). Other risk factors reported only in single studies were housing type (86), increased monthly average temperature (48), increased wind speed (48), increased relative humidity (48), and increased average rainfall (75).

Both studies that utilised HBM identified increased livestock density, land cover, and increased precipitation as associated with LSD occurrence (45, 69). Meanwhile, increased mean temperature (45), increased maximum temperature (69), reduced vapor pressure (69), and reduced wind speed (69) were each reported as risk factors in only one reference. Of the five studies that utilised QRA, three studies concluded that unregulated cattle movement between countries is strongly associated with the risk of LSD entry into countries (57, 78, 85). Two studies modelled the risk of LSD entry into countries through vector introduction via vehicle transportation (77) or wind dispersion across oceans (assuming a maritime crossing of approximately 1,500 km within 48 h) (62). Machine learning algorithms were employed in two studies; one aimed to compare the performance of various algorithms describing the relationship between the occurrence of LSD outbreaks and meteorological and geological factors (42), while the other utilised resampling techniques to test factors influencing the precision of models based on biased data in the context of low numbers of LSD cases (82). Various methods exist for analysing risk factors, but there were differences in the factors reported as statistically significant across studies.

Ten studies utilised various spatiotemporal analyses such as environmental niche modelling (ENM) (44, 45, 47, 69), spatiotemporal cluster analysis (13, 15, 74, 76, 81), and time-series modelling (46). Most of the ENMs identified in these studies were conducted using MaxEnt (44, 45, 69), while one study was performed using Quantum GIS (47). Commonly employed environmental variables in the ENM included land cover (44, 45, 69), livestock density (44, 45, 69), precipitation (44, 45, 47, 69), diurnal range (44, 45), and temperature (45, 47, 69). Isothermality (the ratio of the annual mean diurnal range to the annual temperature range), water vapor pressure, solar radiation, and wind speed were included as environmental variables in the final ENM in the previous studies (47, 69); however, these variables did not include consistently across all final ENMs. Spatiotemporal cluster analysis to identify patterns of disease outbreak was conducted in five studies (13, 15, 74, 76, 81). Two of these studies conducted cluster analyses using various models (space–time permutation, Poisson space–time, and Bernoulli space–time models) on LSD outbreaks that occurred in different regions of Thailand in 2021 (15, 76). Depending on the model chosen, clusters of varying sizes were depicted in different locations. This was also the case in Uganda (74) and Russia (13). The size and location of outbreak clusters may vary depending on regional characteristics and the selected model, so interpreting the results requires consideration of these factors. All five studies exclusively utilised SaTScan™ for spatiotemporal cluster analysis, which may have limitations, such as the inability to consider non-circular clusters (92). Time-series modelling was employed to track trends in the number of LSD outbreak cases by continent, and to forecast future cases (46). These spatiotemporal analyses provide valuable insights into targeted interventions, surveillance, cluster detection, and estimating the number of cases by exploring the spatial and temporal distribution of LSD occurrence.

Twenty-eight studies explored the origin and spread of LSDV using transmission modelling (7–9, 30, 33–39, 44, 51–56, 60, 61, 63, 64, 70–72, 79, 80, 87). Phylogenetic analyses of LSDV typically use the RNA polymerase 30 kDa subunit (RPO30) and the G protein-coupled receptor (GPCR) to compare homology with other strains. Additionally, the EEV glycoprotein gene (33, 37, 39), the p32 envelope protein gene (33, 37), the CaPV homolog of the variola virus B22R (35, 39), and whole genome analysis are also utilised in phylogenetic studies. The results of the phylogenetic analyses of LSDV by country are as follows. The continuous endemic circulation of LSDV was confirmed in Egypt during 2017–2019 (51, 56, 64). The spread of LSDV to Türkiye was inferred to be from Africa and the Middle East (80). LSDV strains collected in north-west China in 2019 shared high homology with vaccine-derived recombinant viruses (87), first reported from Russia in 2017 and later determined to have arisen during seed production of the Lumpivax vaccine (Kenya Veterinary Vaccines Production Institute) that was widely used in a mass vaccination campaign in Kazakhstan in 2016 (93). The LSDV strains from India and Bangladesh in 2019, as well as those from Nepal and Myanmar in 2020, exhibited high sequence identity to each other and were found to be closely related to LSDV strains from Kenya in 1958, rather than to the recombinant vaccine strains from Russia and China (33–35, 39). This suggests a possible common source of infection in South-Asian countries. Tran et al. (36) reported that the p32 and RPO30 genes of the LSDV strain first identified in Vietnam had 100% sequence identity with the Chinese LSDV strain. In Thailand, circulating LSDV strains displayed highest genetic similarity with the recombinant vaccine strains from China and Vietnam, while showing slight molecular differences from the recombinant vaccine strains from Russia (37, 38). Similarly, Indonesian viruses from 2022 clustered with viruses from China, Hong Kong, Taiwan, Bangkok, Vietnam and Thailand collected between 2019 and 2021 (40).

The *R_0_* and *R_E_* were estimated allowing for differences in vector species (60, 79), the progression of the outbreak (54), the first detection time of LSD (71), and variations in livestock production systems (72). Indirect transmission via flying vectors played a significant role in epidemic spread among various transmission methods according to the model, with two studies reporting high *R_0_* values for spread by stable flies (*Stomoxys calcitrans*), mosquitoes (*Aedes aegypti*), and biting midges (*Culicoides nubeculosus*) (60, 79), although LSDV transmission from *Culicoides* sp. to bovines has never been experimentally demonstrated. The median value of *R_0_* in Albania (54) and Türkiye (71) was 0.9, which gradually decreased over time. Molla et al. (72) reported a higher *R_0_* in intensive production systems (estimated *R_0_*, 1.09) than in crop-livestock production systems (estimated *R_0_*, 1.07) in Ethiopia. Alkhamis and VanderWaal (44) calculated the monthly average *R_E_* between 2012 and 2015 as 2.2, indicating continuous spread of LSDV in the Middle East.

Seven studies estimated the transmission speed of LSD using a spatial kernel approach (7, 8, 30, 52, 53, 55, 61). The average transmission distance between farms was estimated to be less than 2.5 km in the four publications (7, 8, 53, 61). However, assuming a maximum value for the kernel parameter and an *R_0_* of 20, the transmission distance between farms can reach up to 80 km (55). The transmission speed by local spatial spread was calculated to be ~1 km/day (30, 52), and all studies mentioned the rapid spread over long distances due to the movement of infected cattle.

EFSA 2017 mapped the transmission path between farms in Türkiye during the outbreak period using transmission network modelling; however, the specific modelling tool used was not mentioned (52). Magori-Cohen et al. (70) estimated a mathematical dynamic transmission model that explained the daily incidence within a farm using transmission rate parameters representing direct and indirect transmission, and transmission via milking; the authors concluded that the daily incidence of LSD within farms was mainly caused by indirect transmission via biting insects. The dynamic transmission modelling performed in three previous studies predicted changes in the susceptible, infected, recovered population of cattle (9, 71, 72). Among these three studies, EFSA 2020 provided a detailed SIR model by additionally incorporating vector life cycle parameters (9). Transmission modelling facilitates inferences around the origins of a disease, its transmissibility, the distance and the sequence of spread, and the contribution of different transmission routes. It provides valuable information and parameters regarding the spread of LSD.

Six studies explored the forward spread of LSD and policy effects through simulation modelling. Four studies utilised scenario modelling (7, 8, 50, 55), while two utilised airborne dispersal and trajectory modelling to track vector dispersion (62, 67). Two scenario modelling studies suggested effective control measures against LSD by forecasting disease spread according to culling criteria and timing, and vaccination policy (7, 8). EFSA (55) helped establish effective vaccination policies through scenario modelling of how long vaccine policies should be implemented depending on vaccine effectiveness and coverage for LSD eradication. Casal et al. (50) employed a dynamic simulation model to estimate and validate the required quantity of vaccine stock in response to the entry of LSD in France, considering certain assumptions. Hall et al. (62) examined the possibility of LSD entry and expected vector distribution in response to climate change. Klausner et al. (67) examined the possibility of LSD-infected vectors invading Israel from Egypt in both 1989 and 2006. Simulation modelling can estimate future changes in population and disease spread and provide effective policies and strategies to prevent spread.

### 3.4 Results of synthesis

Considering the characteristics and objectives of each modelling approach, we categorised modelling approaches that can answer the six key questions we identified to be addressed after the onset of an initial outbreak of LSD (Supplementary Figure S1). Among various sub-categories, machine learning algorithms and time-series modelling were excluded as they are difficult to answer the six questions. We organised the six key questions, using the first occurrence of LSD as the reference point, into three periods: questions about the past, current, and future events in the outbreak. Questions about the past included the origin and entry pathway of LSDV into a country or region, while questions about the current period included the severity of an outbreak and risk factors. Finally, questions about the future included spread and the effectiveness of control measures.

Phylogenetic analyses allow us to use strain evolution to infer the direction of cross-border spread. These analyses play a significant role in understanding the epidemiology of animal diseases, helping to differentiating between endemic and emerging pathogens and aiding in the selection of appropriate vaccine strains (94, 95). These are particularly relevant for rapidly evolving pathogens like influenza and FMD viruses (95). However, for DNA viruses with high genetic stability like LSDV, phylogenetic analyses are less prioritised (96). Moreover, the process of sampling LSDV during an outbreak and analysing genomic sequences can be time-consuming unless prioritised and appropriate systems are in place in advance. Although phylogenetic analysis is highly accurate, it is important to consider limitations such as geographic sampling bias and the public availability of sequences (97). Phylogenetic analyses are useful for inferring evolutionary pathways or comparing strains, but they are not as versatile for non-genetic epidemiological questions (95). Thus, phylogenetic analyses hold a lower priority in the early stages of an LSDV outbreak.

QRA is an essential tool for evaluating the likelihood of animal disease outbreaks and incursions. QRA for LSD was conducted to understand and quantify the potential outcomes and likelihoods of LSD outbreaks, identifying high-risk entry routes and formulating strategies to mitigate these risks (77). QRA can be conducted based on previous data and parameters, thus aiding significantly in the development of strategies to prevent LSDV incursion. During LSD outbreaks, the new outbreak data can be employed to reassess the current risk scenario. The outcomes of QRA are heavily influenced by the parameters used; therefore, the employment of inappropriate or incomplete parameters can lead to biased results and increased uncertainty. Beyond entry route evaluation, QRA is instrumental in assessing various risk factors associated with the transmission of animal diseases and in developing management strategies. Consequently, QRA has been assessed as a moderate priority in responding to LSDV outbreaks.

Airborne dispersal and trajectory modelling enable the tracking of the distribution of LSDV-carrying vector in relation to climatic conditions (62) and their changes (98, 99). This modelling allows for the backward tracing of vector movements from the first outbreak areas to estimate entry points, or forward tracing from the first outbreak areas to estimate potential spread directions (67). Therefore, airborne dispersal and trajectory modelling is an effective tool in addressing questions related to entry pathways and the spread of disease. The urgency of questions regarding entry pathways is considered low because, while this modelling can identify transboundary spread routes, controlling vectors at borders is practically challenging. On the other hand, the urgency of questions related to the spread is considered high. Estimating potential spread routes is crucial for assessing the effectiveness of control measures and facilitating their implementation. Airborne dispersal and trajectory modelling, being a sophisticated model requiring high-quality input data and intensive labour to operate, may take significant time to implement during the outbreak, unless appropriate systems are in place for rapid implementation. Estimating the direction and the spread strength of LSDV-carrying vectors in response to changing climates over time offers valuable insights but also entails a high degree of uncertainty.

The severity and context of a disease outbreak can be understood through descriptive epidemiology, which includes morbidity, mortality, prevalence, epidemic curves, and outbreak mapping. These outputs, which involve simple calculations or visualisations, can be quickly generated and are crucial for immediate public health decisions like resource allocation and emergency response planning. While the results of descriptive epidemiology generally do not contain high uncertainty, they do depend on data quality, emphasising the need for high-quality data acquisition through active surveillance using sensitive and specific detection methods. Monitoring incidence risk after applying control measures tracks changes in new cases, evaluating the efficacy of these measures. Epidemic curves and outbreak mapping can provide useful results such as estimated reproduction number (100), estimated dissemination rates (101), and kernel density map of infected premises (102). Therefore, descriptive epidemiology has been assessed as a high priority.

The risk factors and their contributions to LSD outbreaks contributions can be identified using GLMMs, measures of association, and HBM. Understanding these risk factors is critical for effective disease management and control during LSDV outbreaks, emphasising the importance of these modelling approaches. Prior to an outbreak, existing studies can guide risk factor management. However, risk factors can vary depending on the outbreak context, necessitating efforts to identify country/geography-specific risk factors during the outbreak. This requires accumulated outbreak data from the first occurrence of the disease in a country with ongoing updates to ensure the reliability of identified risk factors. Since disease outbreaks result from multiple interacting factors, some level of uncertainty is inherent in these results. Additionally, the types of questions these methods can answer are limited. Therefore, GLMMs, measures of association, and HBM have been assessed as a moderate priority.

ENM is a tool for identifying zones that are ideally suited for LSDV and its vectors, considering environmental aspects (103). This modelling is instrumental in identifying regions with suitable conditions for LSD introduction and future spread, particularly useful for countries currently free from LSD. These nations can develop ENMs by integrating risk factors from existing research, and developed ENMs can serve as dynamic tools, adaptable with continuous updates during the outbreak. While ENM estimates the potential distribution of LSDV or its vectors, it’s crucial to recognise that LSD does not exclusively occur within the estimated distribution, and not all vectors carry LSDV. Therefore, there is inherent uncertainty in ENM results. Despite these limitations, the ability of ENM to identify potential risk areas for resource allocation makes it a valuable asset in disease management strategies, leading to its assessment as a moderate priority.

The *R_0_* and *R_E_* value are key epidemiological indicators for predicting the potential spread of diseases and the effectiveness of control strategies. These metrics assist in determining the proportion of the population that requires vaccination to achieve herd immunity and in evaluating the real-time effectiveness of control measures (104). Additionally, they are employed in mathematical models to simulate disease spread, enabling them to answer urgent questions, which is why their priority has been considered high. While R*_0_* and R*_E_* can be calculated quickly, the accuracy of the results may vary depending on the assumptions of the estimators and the precision of the data (105).

Spatial kernel-based transmission rate estimation not only aids in investigating the spread range from infected premises and implementing corresponding control measures, but also, as the estimated spatial kernels are incorporated into simulations of epidemic spread, there is an urgent need to address this modelling in the early stages of an LSD outbreak (106). Spatial kernel estimation requires accumulated data, but it can also be empirically estimated based on insights from previous studies. However, given the limited scope of questions this modelling can answer and the inherent uncertainties, this modelling has been assigned a moderate level of priority.

Dynamic transmission modelling is valuable for investigating patterns and behaviours of disease spread within populations over time. The construction of these models requires accumulated data. Dynamic transmission modelling, which enables the exploration and prediction of disease trends, is somewhat urgently needed in the early stages of an LSD outbreak. However, dynamic transmission modelling entails high uncertainty due to the estimation of parameters based on observed data and the subsequent prediction of new infections. Consequently, dynamic transmission modelling has been assessed as having a moderate priority.

Transmission network modelling is for analysing transmission networks between premises. Transmission network modelling is created based on accumulated data and provides limited answers, such as identifying who infected whom. While the uncertainty of transmission network modelling can be reduced with well-prepared data, obtaining detailed and accurate contact tracing and surveillance data during the early stages of an LSD outbreak is challenging, leading to a low prioritisation of transmission network modelling (107).

Scenario modelling enables the estimation of changes in the infected population within potential scenarios of LSD spread and control measures. This modelling can be applied in various aspects. Some previous studies have assessed how the implementation of control measures could alter the dynamics of an LSD outbreak (7, 8). Others have estimated the necessary duration of vaccination policies and required vaccine stock quantities under various scenarios of vaccine coverage and efficacy (50, 55). The development of scenario modelling requires a considerable amount of data and labour, dependent on the complexity of the scenario’s assumptions and the accuracy of included parameters. The results of scenario modelling carry a high level of uncertainty due to these assumptions and the current progress of the disease outbreak. Despite these challenges, versatile scenario modelling can support key decision-making processes such as strategy development and resource allocation following the initial outbreak of LSD. Therefore, scenario modelling has to be urgently addressed in the early response stages and is considered a high priority.

### 3.5 Modelling workflow

Based on the existing literature, we propose the following modelling workflow to address the first LSD occurrence in a country. In planning for a potential LSD outbreak in a country, preliminary analyses may be conducted using existing international outbreak data and estimated parameters. QRA can be used to assess LSD incursion risk, while GLMMs, measures of association, and HBM can be employed to identify risk factors and their relative contributions in past outbreaks. ENM can then be used to identify areas highly suitable for LSDV presence, supporting decision-making for preventive policies against LSD. Additionally, preparatory work can be carried out for models that will be immediately activated upon outbreak confirmation. This includes setting up systems for handling genomic and epidemiological data, creating visualisations, and establishing analysis routines (i.e., data pipelines).

Descriptive epidemiology is quickly carried out following the first LSD occurrence to determine the severity and context of the current outbreak using data as they become available. Using outbreak data, clusters of cases are detected, and the connections between these clusters are tracked through the monitoring of animal movements and vector distributions (and perhaps phylogenetic analyses). Information on risk factors can be updated using GLMMs, measures of association, and HBM based on data from rapidly conducted epidemiological studies in the affected country. ENM and QRA results can be updated based on the newly identified risk factors and information from field investigations, assisting in making informed decisions to mitigate the outbreak situation. Phylogenetic analyses may be used to identify the origin of an LSDV outbreak, helping to ensure that further introductions are prevented, and to inform selection of effective vaccines.

After sufficient data have been obtained following the first detection of an LSD outbreak, the severity and potential direction of spread can be assessed using *R_0_*, *R_E_*, spatial kernel-based transmission rate estimation, and airborne dispersion and trajectory modelling. These analyses support decision-making for LSDV control policy implementation. When scenario modelling becomes available, estimating the progression of the LSD outbreak in various scenarios and carefully reviewing them enables the selection of appropriate LSDV control measures. Dynamic transmission modelling and transmission network modelling may inform the parameterisation of scenario modelling or be utilised for back-, now-, and fore-casting, and also be employed to rapidly evaluate the effectiveness of implemented measures for LSDV control.

Above all, to build a model that reflects reality and supports informed decision-making, an accurate and precise dataset is essential. By identifying the necessary information set for each key stage of the outbreak and ensuring that information is continuously updated as the outbreak progresses, it is possible to provide updated parameters to inform sophisticated simulation models. This approach can enable models to guide informed decision-making, help overcome information bias, and reduce modelling uncertainty by continuously updating and providing valuable information.

## 4 Discussion

LSD is a transboundary disease, and the current rapid spread of the disease from Africa to the Middle East, Eurasia, and Southeast Asia indicates the potential for further global spread (62). Although scientists and decision-makers will collaborate to discuss the direction of disease control and prevention and provide swift responses after the first occurrence in a country or region, the available modelling types for responding to LSD outbreaks and information regarding their timeliness are currently lacking. This systematic review was conducted to identify applicable modelling tools that may assist in the response to an LSD outbreak, examine their timeliness, and develop a workflow to be performed incorporating prioritisation based on the available data, urgency, model preparation period, uncertainty, and limitations.

Applicable models were grouped into five analysis types and 17 sub-categories (Figure 1). The final table for timeliness of modelling included 14 sub-categories due to the constraints of some modelling (included types: basic statistics, epidemic curve and outbreak mapping, QRA, GLMMs, measures of association, HBM, ENM, phylogenetics, *R_0_* and *R_E_* estimation, spatial kernel-based transmission rate estimation, dynamic transmission modelling, transmission network modelling, scenario modelling, and airborne dispersion and trajectory modelling). These types of analyses and their sub-categories have been classified into three time points based on when modelling can address specific questions, as indicated in Supplementary Figure S1 (i.e., past events, current events, and future events). Additionally, they have been categorised into three different phases according to modelling timeliness, as outlined in the modelling workflow in the results section (i.e., pre-outbreak, early outbreak phase from day 0 to 7, and late outbreak phase spanning weeks to months). In this discussion, we have addressed them in the latter sequence.

The following eight modelling approaches—QRA, airborne dispersion and trajectory modelling, GLMMs, measures of association, HBM, ENM, spatial kernel-based transmission rate, and simulation modelling—can be conducted as a preliminary analysis before an LSD outbreak, providing the context of LSD spread and potential risk factors of the entry of LSD. The major limitation of QRA is that it is often based on incomplete data or uncertain assumptions, which can affect the accuracy of the risk assessment (108). Airborne dispersion and trajectory modelling can be used to estimate the spread of vectors between areas, thereby identifying potential pathways of LSD entry (62). Airborne dispersion and trajectory modelling are designed under the assumption that the dispersion of viruses and vectors is similar to particle dispersion. As a result, uncertainty is introduced into the outcomes of the modelling (109, 110). Additionally, airborne dispersion and trajectory modelling relies on weather and atmospheric data with high level of uncertainty, and not all vectors carry LSDV, further contributing to uncertainties (62, 111). GLMMs, measures of association, and HBM can be used to identify risk factors that contribute to LSD outbreaks. However, these risk factors were inconsistent between studies, which could have been influenced by the diverse climate and environments of the assessed countries (48, 58, 63, 66, 73, 75, 80, 86). ENM provides information on areas that could be targeted for surveillance before LSD entry, but the accuracy of the results depends on up-to-date and accurate data on environmental variables (112).

The spatial kernel-based transmission rate is typically derived from previous outbreak data in other countries, which makes it challenging to apply directly to new outbreak areas until sufficient data is available for its estimation in the local context (7, 8, 30, 52, 53, 55, 61). While no instances of pre-outbreak simulation modelling for LSD were found in the relevant literature, simulation modelling can be employed for pre-planning and preparedness against transboundary diseases like FMD. AusSpread, a regional model developed within a Geographic Information System environment, simulates disease spread and control across various regions of the Australian continent, encompassing diverse environments and production systems (113). The Australian Animal Disease Spread Model (AADIS), an extension built upon key features of AusSpread, models five independent spread methods of FMD transmission within herds, illustrating the paths of infection on a map (114). While the epidemiological unit of interest of AusSpread is a farm, AADIS utilises herds within farms as its epidemiological unit of interest. Therefore, AADIS captures the heterogeneity of production systems that manage different species or production classes within farms. By crafting plausible outbreak scenarios before an LSD outbreak and simulating the spread of disease within herds and between farms, the potential repercussions of an LSD outbreak can be estimated, facilitating the formulation of control measures and contingency plans.

At the early phase of LSD outbreak (from day 0 to 7), the following nine modelling approaches can be employed: descriptive epidemiology, QRA, GLMMs, measures of association, HBM, ENM, spatial kernel-based transmission rate, dynamic transmission modelling, and transmission network modelling. By identifying the urgency of the situation and areas that require immediate action, descriptive epidemiology can support decision-making shortly after detection of LSD. The remaining eight models require a certain amount of accumulated outbreak data; these can be applied from as early as 1 week after the first detection of LSD. After the first detection of LSD, accurate and systematic data collection and management become essential to analyse and prevent disease spread. It is necessary to minimise bias and errors during data collection to maintain high data quality, and a standardised data format should be applied to ensure efficient management of the data. Data preprocessing is the most time-consuming part of the analysis process, and using a standardised data format can reduce analysis time and provide rapid results (115). Therefore, collecting accurate data and organising it in a centralised and standardised format not only enables the immediate implementation of descriptive epidemiology and risk factor analysis models but also facilitates the prompt application of control measures and higher-level models.

Phylogenetic analyses, *R_0_* and *R_E_* estimation, airborne dispersal and trajectory modelling, and scenario modelling can be conducted once sufficient data has been collected, or when the parameters necessary for the models have been calculated (weeks to months). These modelling approaches facilitate evidence-based decision-making in disease control and provide valuable insights into disease spread, infectiousness, and the effectiveness of control measures. These modelling approaches have been widely developed and applied across various diseases. In prior studies, researchers applied airborne dispersion and trajectory modelling to estimate the potential incursion of biting midges concerning the spread of bluetongue virus (99, 116). For FMD, diverse scenario models (such as Exodis-FMD™, AADIS, and InterSpread+) were developed to estimate disease spread and establish the foundation for preventive measures and response strategies (117). While phylogenetic analyses, *R_0_* and *R_E_* estimation, and airborne dispersal and trajectory modelling can be executed relatively quickly when the required data become available, implementing simulation modelling for LSD may take over a month due to the complexity of parameters and hypotheses involved in its application.

In summary, the eight models mentioned above for use prior to an outbreak provide information that identifies potential entry pathways and risk factors, determines high-risk areas for spread within a country, and estimates the scale of spatiotemporal spread. Based on this information and rationale, enhanced quarantine measures for potential entry pathways and early surveillance of high-risk areas can be implemented. Additionally, preventive plans and control measures can be established, including providing guidelines to livestock owners in high-risk areas, setting up temporary emergency reporting networks, and determining the radius of ring vaccination or culling. We can better prepare for future LSD outbreaks in a country through these measures. However, the aforementioned eight models may be highly dependent on the outbreak context and therefore are not always generalisable, potentially making them less suitable for direct application in countries that have previously been free from LSD. Therefore, to effectively respond to future LSD outbreaks, it is crucial to proactively prepare and establish models based on the full range of literature parameters prior to an outbreak, while accounting for uncertainty.

Following an LSD outbreak, updated outbreak data should be utilised to identify relevant risk factors, and regular follow-up should be ensured to make informed decisions. In the early stages of an LSD outbreak, modelling focuses on understanding the spatiotemporal dynamics of spread and identifying risk factors specific to the country. This enables the determination of when and where to apply emergency responses and allows for the control and hygienic disposal of the specific risk factors. Emergency responses may include stamping out, movement restrictions, vaccination, enhanced biosecurity, zoning or compartmentalisation of infected areas, cleaning, disinfection, and application of insecticides (6). The extent and priority of these emergency responses may vary depending on national resources. In the later stages of an LSD outbreak, more complex methodologies can be employed to track and project the spatiotemporal spread and the effectiveness of interventions. Eradication of disease from infected areas is a long-term project. The results from modelling can inform which interventions are effective for medium- to long-term application and aid in the development of future strategies and measures.

Optimising parameters and developing simplified versions of scenario models during their implementation is necessary to enable rapid assessment and understanding of the unfolding epidemic and assist with control. Parameter optimisation enables more accurate forecasting and informed decision-making based on reliable information by reducing estimation errors and uncertainties and facilitating model generalisation (118, 119). Developing simplified scenario models can support rapid decision-making during the early phases of an LSD outbreak, by virtue of their simplicity and ability to be rapidly updated as new information comes in. Although these models may have lower accuracy, their speed and agility can make them useful tools for quickly responding to the spread of LSD (120). In summary, parameter optimisation and the development of simplified versions of scenario models are crucial for rapidly forecasting LSD spread and supporting outbreak management during the early stages. Afterward, the application of more complex and advanced scenario models can provide more accurate results, thereby aiding in the best decision-making for controlling the initially managed outbreak situation.

Our modelling workflow can be broadly applied to various diseases with some modifications, making it a first step in preparing for disease introduction into a country. The workflow focuses on models specific to LSD, and therefore, modelling implemented for other diseases was not considered. To further strengthen the workflow, it would be beneficial to incorporate modelling that can be extended through a systematic review of modelling for other animal diseases. The priorities and time required for modelling implementation can vary. The priorities presented in the results were assessed based on four criteria. Among them, we placed greater importance on the urgency of the questions that the model can address during an LSD outbreak. Therefore, these priorities can change depending on which criteria are given more importance. If the necessary resources and programs are prepared in advance, modelling implementation can be completed quickly; otherwise, it may take several weeks to months. For example, the time required for phylogenetic analyses can be shortened if sufficient representative sequences are publicly available (121). The implementation time for airborne dispersion and trajectory modelling can be reduced by establishing prior agreements with meteorological agencies to obtain the necessary meteorological data. The implementation could be delayed accordingly if more time is required for programme preparation or stabilisation.

The proposed modelling workflow incorporates resource-intensive approaches that are beneficial in resource-rich environments. However, these modelling approaches may be challenging to implement directly in resource-limited settings. In particular, low-resource countries with limited personnel and budget may predominantly concentrate on direct disease response and control during LSD outbreaks with limited capacity for the application of secondary data processing and modelling. In such scenarios, if LSD outbreaks occur without adequately informed decision-making and control measures, significant economic losses and financial burdens on households are likely to be unavoidable (122). Consequently, it is recommended that countries with limited resources focus more on disease prevention strategies. These nations should prioritise the establishment of robust biosecurity policies and early detection and reporting systems (32) informed by the pre-outbreak modelling workflow proposed in this paper, such as estimating LSD entry pathways through QRA and preparing models that can be activated immediately upon outbreak.

The models included in Supplementary Figure S1 generally involve considerable uncertainty, and the sources of uncertainty vary depending on the modelling approaches (123). Failing to account for uncertainty in modelling ultimately leads to various issues, including inaccurate projections, incorrect decisions, and the inability to estimate the actual effects of interventions. To address these issues, various methods have been proposed, including data assimilation, sensitivity analysis, model validation and verification, and uncertainty quantification (119, 123–125). The use of data assimilation enables real-time application of dynamic observational data to the model, and sensitivity analysis can be performed to better understand the influence of highly variable or uncertain input parameters on model outputs. By reducing uncertainty in modelling, we can develop more accurate and reliable models to support timely decision-making in response to LSD outbreaks. Ultimately, this will improve our ability to prevent and control the spread of the disease. However, the uncertainty of modelling is inevitable. Therefore, analytical results and their meanings including uncertainty should be communicated to decision-makers avoiding potential for misunderstanding (126). Additionally, modelling findings should never be used in isolation, and should always be interpreted in conjunction with field data and observations.

## 5 Conclusion

In this review we found descriptive epidemiology studies to be overrepresented, while simulation modelling was underrepresented. Descriptive epidemiology can be immediately leveraged during the early stages of LSD outbreaks to provide timely support for decision-making, although it may only provide limited information about the severity of an outbreak in its early phase. To support decision-making in subsequent phases, simulation modelling can be used to evaluate the future spread potential, impacts and effectiveness of counter measures. Because the implementation of complex simulation modelling is challenging to undertake in a timely manner, there is a need to fit and use cruder simulation models that can support outbreak decision-making. Our modelling workflow offers a structured approach to employ high-quality modelling tailored to various phases of transboundary disease incursion, such as LSD, with potential applicability to other diseases. Though, it is recognised that many approaches are resource intensive, which may limit their feasibility in resource-poor environments. Critically, ensuring high data quality and considering uncertainty when interpreting and communicating model results are imperative.

## Data Availability

The original contributions presented in the study are included in the article/Supplementary material, further inquiries can be directed to the corresponding author.

## References

[1] Al-Salihi K . Lumpy skin disease: review of literature. Mirror Res Vet Sci Anim. (2014) 3:6–23.

[2] Grubman MJ Baxt B. Foot-and-mouth disease. Clin Microbiol Rev. (2004) 17:465–93. doi: 10.1128/CMR.17.2.465-493.2004, PMID: 15084510 PMC387408

[3] Davies G . The foot and mouth disease (FMD) epidemic in the United Kingdom 2001. Comp Immunol Microbiol Infect Dis. (2002) 25:331–43. doi: 10.1016/S0147-9571(02)00030-9, PMID: 12365809

[4] Wilkinson K Lowe P Donaldson A. Beyond policy networks: policy framing and the politics of expertise in the 2001 foot and mouth disease crisis. Public Adm. (2010) 88:331–45. doi: 10.1111/j.1467-9299.2010.01831.x 20726153

[5] Animal Health Australia . AUSVETPLAN: Overview (version 5.0). Edition 5. (2021). Available at: https://animalhealthaustralia.com.au/ausvetplan (accessed July 3, 2024).

[6] Animal Health Australia . Response strategy: Lumpy skin disease (version 5.0). Edition 5. (2022). Available at: https://animalhealthaustralia.com.au/wp-content/uploads/2022/08/AUSVETPLAN-Manuals_Response_Lumpy-skin-disease.pdf (accessed July 3, 2024).

[7] EFSA AHAW Panel (EFSA Panel on Animal Health and Welfare) . Scientific opinion on lumpy skin disease. EFSA J. (2015) 13:3986. doi: 10.2903/j.efsa.2015.3986

[8] EFSA AHAW Panel (EFSA Panel on Animal Health and Welfare) . Urgent advice on lumpy skin disease. EFSA J. (2016) 14:4573. doi: 10.2903/j.efsa.2016.4573

[9] EFSA (European Food Safety Authority) Calistri P De Clercq K Gubbins S Klement E Stegeman A . Lumpy skin disease epidemiological report IV: data collection and analysis. EFSA J. (2020) 18:e06010. doi: 10.2903/j.efsa.2020.6010, PMID: 32874220 PMC7448019

[10] Ferguson NM Cummings DAT Cauchemez S Fraser C Riley S Meeyai A . Strategies for containing an emerging influenza pandemic in Southeast Asia. Nature. (2005) 437:209–14. doi: 10.1038/nature04017, PMID: 16079797

[11] Haegeman A Sohier C Mostin L De Leeuw I Van Campe W Philips W . Evidence of lumpy skin disease virus transmission from subclinically infected cattle by *Stomoxys calcitrans*. Viruses. (2023) 15:1285. doi: 10.3390/v15061285, PMID: 37376585 PMC10301266

[12] Sprygin A Pestova Y Wallace DB Tuppurainen E Kononov AV. Transmission of lumpy skin disease virus: a short review. Virus Res. (2019) 269:197637. doi: 10.1016/j.virusres.2019.05.015 31152757

[13] Byadovskaya O Prutnikov P Shalina K Babiuk S Perevozchikova N Korennoy F . The changing epidemiology of lumpy skin disease in Russia since the first introduction from 2015 to 2020. Transbound Emerg Dis. (2022) 69:e2551–62. doi: 10.1111/tbed.14599 35583857

[14] Sanz-Bernardo B Haga IR Wijesiriwardana N Hawes PC Simpson J Morrison LR . Lumpy skin disease is characterized by severe multifocal dermatitis with necrotizing fibrinoid vasculitis following experimental infection. Vet Pathol. (2020) 57:388–96. doi: 10.1177/0300985820913268, PMID: 32314676 PMC7201124

[15] Arjkumpa O Suwannaboon M Boonrod M Punyawan I Liangchaisiri S Laobannue P . The first lumpy skin disease outbreak in Thailand (2021): epidemiological features and spatio-temporal analysis. Front Vet Sci. (2021) 8:799065. doi: 10.3389/fvets.2021.799065, PMID: 35071388 PMC8782428

[16] Page MJ McKenzie JE Bossuyt PM Boutron I Hoffmann TC Mulrow CD . The PRISMA 2020 statement: an updated guideline for reporting systematic reviews. Int J Surg. (2021) 88:105906. doi: 10.1016/j.ijsu.2021.105906, PMID: 33789826

[17] Moher D Shamseer L Clarke M Ghersi D Liberati A Petticrew M . Preferred reporting items for systematic review and meta-analysis protocols (PRISMA-P) 2015 statement. Syst Rev. (2015) 4:1. doi: 10.1186/2046-4053-4-1, PMID: 25554246 PMC4320440

[18] Peterson AT Soberón J. Species distribution modeling and ecological niche modeling: getting the concepts right. Nat Conserv. (2012) 10:102–7. doi: 10.4322/natcon.2012.019

[19] Cohen T White P. Transmission-dynamic models of infectious diseases. In: Abubakar I Stagg HR Cohen T Rodrigues LC, editors. Infectious disease epidemiology. Oxford: Oxford University Press (2016). 223–42.

[20] Kasereka SK Zohinga GN Kiketa VM Ngoie RBM Mputu EK Kasoro NM . Equation-based modeling vs. agent-based modeling with applications to the spread of COVID-19 outbreak. Mathematics. (2023) 11:253. doi: 10.3390/math11010253

[21] Mazloum A Van Schalkwyk A Babiuk S Venter E Wallace DB Sprygin A. Lumpy skin disease: history, current understanding and research gaps in the context of recent geographic expansion. Front Microbiol. (2023) 14:1266759. doi: 10.3389/fmicb.2023.1266759 38029115 PMC10652407

[22] Weiss KE . Lumpy skin disease virus. In: Gard S Hallauer C Meyer KF, editors. Rinderpest Virus Lumpy Skin Disease Virus. Berlin Heidelberg: Springer (1968). 111–31.

[23] Abebaw B . Prevalence of lumpy skin disease in Africa: a systematic review and meta-analysis from 2007 to 2023. Vet Med Int. (2024) 2024:9991106. doi: 10.1155/2024/9991106, PMID: 38868352 PMC11168804

[24] Davies FG . Lumpy skin disease of cattle: a growing problem in Africa and the near East. World Anim Rev. (1991) 68:37–42.

[25] Davies FG . Lumpy skin disease, an African capripox virus disease of cattle. Br Vet J. (1991) 147:489–503. doi: 10.1016/0007-1935(91)90019-J 1777792

[26] Namazi F Khodakaram TA. Lumpy skin disease, an emerging transboundary viral disease: a review. Vet Med Sci. (2021) 7:888–96. doi: 10.1002/vms3.434, PMID: 33522708 PMC8136940

[27] Beard PM . Lumpy skin disease: a direct threat to Europe. Vet Rec. (2016) 178:557–8. doi: 10.1136/vr.i2800, PMID: 27235496

[28] Abutarbush SM Ababneh MM Al Zoubi IG Al Sheyab OM Al Zoubi MG Alekish MO . Lumpy skin disease in Jordan: disease emergence, clinical signs, complications and preliminary-associated economic losses. Transbound Emerg Dis. (2015) 62:549–54. doi: 10.1111/tbed.12177, PMID: 24148185

[29] OIE-WAHIS . Events management. Available at: https://wahis.woah.org/#/event-management (accessed Aug 8, 2024).

[30] Mercier A Arsevska E Bournez L Bronner A Calavas D Cauchard J . Spread rate of lumpy skin disease in the Balkans, 2015–2016. Transbound Emerg Dis. (2018) 65:240–3. doi: 10.1111/tbed.12624 28239954

[31] Tuppurainen ESM Oura CAL. Review: lumpy skin disease: an emerging threat to Europe, the Middle East and Asia. Transbound Emerg Dis. (2012) 59:40–8. doi: 10.1111/j.1865-1682.2011.01242.x, PMID: 21749675

[32] Akther M Akter SH Sarker S Aleri JW Annandale H Abraham S . Global burden of lumpy skin disease, outbreaks, and future challenges. Viruses. (2023) 15:1861. doi: 10.3390/v15091861, PMID: 37766268 PMC10535115

[33] Sudhakar SB Mishra N Kalaiyarasu S Jhade SK Hemadri D Sood R . Lumpy skin disease (LSD) outbreaks in cattle in Odisha state, India in august 2019: epidemiological features and molecular studies. Transbound Emerg Dis. (2020) 67:2408–22. doi: 10.1111/tbed.13579 32304275

[34] Badhy SC Chowdhury MGA Settypalli TBK Cattoli G Lamien CE Fakir MAU . Molecular characterization of lumpy skin disease virus (LSDV) emerged in Bangladesh reveals unique genetic features compared to contemporary field strains. BMC Vet Res. (2021) 17:61. doi: 10.1186/s12917-021-02751-x, PMID: 33514360 PMC7844896

[35] Koirala P Meki IK Maharjan M Settypalli BK Manandhar S Yadav SK . Molecular characterization of the 2020 outbreak of lumpy skin disease in Nepal. Microorganisms. (2022) 10:539. doi: 10.3390/microorganisms10030539, PMID: 35336114 PMC8954389

[36] Tran HTT Truong AD Dang AK Ly DV Nguyen CT Chu NT . Lumpy skin disease outbreaks in Vietnam, 2020. Transbound Emerg Dis. (2021) 68:977–80. doi: 10.1111/tbed.14022, PMID: 33548101

[37] Suwankitwat N Songkasupa T Boonpornprasert P Sripipattanakul P Theerawatanasirikul S Deemagarn T . Rapid spread and genetic characterisation of a recently emerged recombinant lumpy skin disease virus in Thailand. Vet Sci. (2022) 9:542. doi: 10.3390/vetsci9100542, PMID: 36288155 PMC9609959

[38] Sariya L Paungpin W Chaiwattanarungruengpaisan S Thongdee M Nakthong C Jitwongwai A . Molecular detection and characterization of lumpy skin disease viruses from outbreaks in Thailand in 2021. Transbound Emerg Dis. (2022) 69:e2145–52. doi: 10.1111/tbed.14552, PMID: 35396931

[39] Maw MT Khin MM Hadrill D Meki IK Settypalli TBK Kyin MM . First report of lumpy skin disease in Myanmar and molecular analysis of the field virus isolates. Microorganisms. (2022) 10:897. doi: 10.3390/microorganisms10050897, PMID: 35630342 PMC9143258

[40] Sendow I Meki IK Dharmayanti NLPI Hoerudin H Ratnawati A Settypalli TBK . Molecular characterization of recombinant LSDV isolates from 2022 outbreak in Indonesia through phylogenetic networks and whole-genome SNP-based analysis. BMC Genomics. (2024) 25:240. doi: 10.1186/s12864-024-10169-6, PMID: 38438878 PMC10913250

[41] Abera Z Degefu H Gari G Kidane M. Sero-prevalence of lumpy skin disease in selected districts of west Wollega zone, Ethiopia. BMC Vet Res. (2015) 11:135. doi: 10.1186/s12917-015-0432-7 26082259 PMC4468805

[42] Afshari SE . Assessing machine learning techniques in forecasting lumpy skin disease occurrence based on meteorological and geospatial features. Trop Anim Health Prod. (2022) 54:55:55. doi: 10.1007/s11250-022-03073-2, PMID: 35029707 PMC8759057

[43] Alemayehu G Leta S Eshetu E Mandefro A. Incidence of lumpy skin disease and associated risk factors among export-oriented cattle feedlots at Adama District, Central Ethiopia. J Vet Med Anim Health. (2015) 7:128–34. doi: 10.5897/JVMAH2014

[44] Alkhamis MA VanderWaal K. Spatial and temporal epidemiology of lumpy skin disease in the Middle East, 2012–2015. Front Vet Sci. (2016) 3:19. doi: 10.3389/fvets.2016.00019, PMID: 26973845 PMC4776163

[45] Allepuz A Casal J Beltrán-Alcrudo D. Spatial analysis of lumpy skin disease in Eurasia-predicting areas at risk for further spread within the region. Transbound Emerg Dis. (2019) 66:813–22. doi: 10.1111/tbed.13090 30520550

[46] Anwar A Na-Lampang K Preyavichyapugdee N Punyapornwithaya V. Lumpy skin disease outbreaks in Africa, Europe, and Asia (2005–2022): multiple change point analysis and time series forecast. Viruses. (2022) 14:2203. doi: 10.3390/v14102203 36298758 PMC9611638

[47] Ardestani EG Mokhtari A. Modeling the lumpy skin disease risk probability in central Zagros Mountains of Iran. Prev Vet Med. (2020) 176:104887. doi: 10.1016/j.prevetmed.2020.104887, PMID: 32032798

[48] Abdelgawad SET . Establishing econometric modeling equations for lumpy skin disease outbreaks in the Nile Delta of Egypt under current climate conditions. Int J Anim Vet Sci. (2017) 11:260–63. doi: 10.5281/zenodo.1129702

[49] Bouchemla F Agoltsov VA Larionov SV Popova OM Shvenk EV. Epizootiological study on spatiotemporal clusters of Schmallenberg virus and lumpy skin diseases: the case of Russia. Vet World. (2018) 11:1229–36. doi: 10.14202/vetworld.2018.1229-1236 30410226 PMC6200570

[50] Casal J Saegerman C Bertagnoli S Meyer G Ganière JP Caufour P . A simple method to estimate the number of doses to include in a bank of vaccines. The case of lumpy skin disease in France. PLoS One. (2019) 14:e0210317. doi: 10.1371/journal.pone.0210317 30682041 PMC6347152

[51] Diab HM Ahmed AS Batiha GES Alkazmi L El-Zamkan MA. Molecular surveillance of lumpy skin disease outbreak, 2019 in Sohag, Egypt: enzootic potential, phylogenetic assessment and implications on cattle herds health. J Anim Health Prod. (2021) 9:406–16. doi: 10.17582/journal.jahp/2021/9.4.406.416

[52] EFSA (European food safety authority) . Lumpy skin disease: I. Data collection and analysis. EFSA J. (2017) 15:e04773. doi: 10.2903/j.efsa.2017.4773, PMID: 32625471 PMC7010117

[53] EFSA (European food safety authority) . Lumpy skin disease II. Data collection and analysis. EFSA J. (2018) 16:e05176. doi: 10.2903/j.efsa.2018.5176, PMID: 32625811 PMC7009581

[54] EFSA (European Food Safety Authority) Calistri P DeClercq K Gubbins S Klement E Stegeman A . Lumpy skin disease: III. Data collection and analysis. EFSA J. (2019) 17:e05638. doi: 10.2903/j.efsa.2019.5638, PMID: 32626261 PMC7009259

[55] EFSA (European Food Safety Authority) Calistri P DeClercq K De Vleeschauwer A Gubbins S Klement E . Lumpy skin disease: scientific and technical assistance on control and surveillance activities. EFSA J. (2018) 16:e05452. doi: 10.2903/j.efsa.2018.5452, PMID: 32625728 PMC7009741

[56] El-Ansary RE El-Dabae WH Bream AS El Wakil A. Isolation and molecular characterization of lumpy skin disease virus from hard ticks, Rhipicephalus (Boophilus) annulatus in Egypt. BMC Vet Res. (2022) 18:302. doi: 10.1186/s12917-022-03398-y, PMID: 35932057 PMC9354321

[57] Farra D De Nardi M Lets V Holopura S Klymenok O Stephan R . Qualitative assessment of the probability of introduction and onward transmission of lumpy skin disease in Ukraine. Microb Risk Anal. (2022) 20:100200. doi: 10.1016/j.mran.2021.100200

[58] Gari G Waret-Szkuta A Grosbois V Jacquiet P Roger F. Risk factors associated with observed clinical lumpy skin disease in Ethiopia. Epidemiol Infect. (2010) 138:1657–66. doi: 10.1017/S0950268810000506 20233495

[59] Gari G Grosbois V Waret-Szkuta A Babiuk S Jacquiet P Roger F. Lumpy skin disease in Ethiopia: seroprevalence study across different agro-climate zones. Acta Trop. (2012) 123:101–6. doi: 10.1016/j.actatropica.2012.04.009 22569562

[60] Gubbins S . Using the basic reproduction number to assess the risk of transmission of lumpy skin disease virus by biting insects. Transbound Emerg Dis. (2019) 66:1873–83. doi: 10.1111/tbed.13216, PMID: 31038286 PMC6767157

[61] Gubbins S Stegeman A Klement E Pite L Broglia A Cortiñas AJ. Inferences about the transmission of lumpy skin disease virus between herds from outbreaks in Albania in 2016. Prev Vet Med. (2020) 181:104602. doi: 10.1016/j.prevetmed.2018.12.008, PMID: 30581093 PMC7456782

[62] Hall R Torpy J Nye R Zalcman E Cowled B. Quantitative risk assessment for the introduction of lumpy skin disease virus into Australia via non-regulated pathways. Canberra, Australia: AusVet Pty ltd.; (2022). Report no.: C08233. Available at: https://www.agriculture.gov.au/sites/default/files/documents/ausvet-lsd-quantitative-assessment.pdf (accessed July 3, 2024).

[63] Hasib FMY Islam MS Das T Rana EA Uddin MH Bayzid M . Lumpy skin disease outbreak in cattle population of Chattogram, Bangladesh. Vet Med Sci. (2021) 7:1616–24. doi: 10.1002/vms3.524 33993641 PMC8464269

[64] Hodhod A Elgendy E Abd El-Moniem MI Ibrahim MS. Isolation and molecular characterization of lumpy skin disease virus in Egypt during 2017–2018. Eur J Pharm Med Res. (2020) 7:96–103.

[65] Kahana-Sutin E Klement E Lensky I Gottlieb Y. High relative abundance of the stable fly *Stomoxys calcitrans* is associated with lumpy skin disease outbreaks in Israeli dairy farms. Med Vet Entomol. (2017) 31:150–60. doi: 10.1111/mve.12217 27976815

[66] Kiplagat SK Kitala PM Onono JO Beard PM Lyons NA. Risk factors for outbreaks of lumpy skin disease and the economic impact in cattle farms of Nakuru County. Front Vet Sci. (2020) 7:259. doi: 10.3389/fvets.2020.00259 32548130 PMC7274042

[67] Klausner Z Fattal E Klement E. Using synoptic systems’ typical wind trajectories for the analysis of potential atmospheric long-distance dispersal of lumpy skin disease virus. Transbound Emerg Dis. (2017) 64:398–410. doi: 10.1111/tbed.12378 26011073

[68] Lu G Xie J Luo J Shao R Jia K Li S. Lumpy skin disease outbreaks in China, since 3 august 2019. Transbound Emerg Dis. (2021) 68:216–9. doi: 10.1111/tbed.13898 33119963

[69] Machado G Korennoy F Alvarez J Picasso-Risso C Perez A VanderWaal K. Mapping changes in the spatiotemporal distribution of lumpy skin disease virus. Transbound Emerg Dis. (2019) 66:2045–57. doi: 10.1111/tbed.13253 31127984

[70] Magori-Cohen R Louzoun Y Herziger Y Oron E Arazi A Tuppurainen E . Mathematical modelling and evaluation of the different routes of transmission of lumpy skin disease virus. Vet Res. (2012) 43:1. doi: 10.1186/1297-9716-43-1, PMID: 22236452 PMC3268087

[71] Mat B Arikan MS Akin AC Çevrimli MB Yonar H Tekindal MA. Determination of production losses related to lumpy skin disease among cattle in Turkey and analysis using SEIR epidemic model. BMC Vet Res. (2021) 17:300. doi: 10.1186/s12917-021-02983-x, PMID: 34493272 PMC8425146

[72] Molla W Frankena K MCM DJ. Transmission dynamics of lumpy skin disease in Ethiopia. Epidemiol Infect. (2017) 145:2856–63. doi: 10.1017/S0950268817001637, PMID: 28768560 PMC9203443

[73] Molla W Frankena K Gari G Kidane M Shegu D de Jong MCM. Seroprevalence and risk factors of lumpy skin disease in Ethiopia. Prev Vet Med. (2018) 160:99–104. doi: 10.1016/j.prevetmed.2018.09.029 30389003

[74] Ochwo S VanderWaal K Munsey A Ndekezi C Mwebe R Okurut ARA . Spatial and temporal distribution of lumpy skin disease outbreaks in Uganda (2002–2016). BMC Vet Res. (2018) 14:174. doi: 10.1186/s12917-018-1503-3, PMID: 29859091 PMC5984736

[75] Ochwo S VanderWaal K Munsey A Nkamwesiga J Ndekezi C Auma E . Seroprevalence and risk factors for lumpy skin disease virus seropositivity in cattle in Uganda. BMC Vet Res. (2019) 15:236. doi: 10.1186/s12917-019-1983-9, PMID: 31286926 PMC6615106

[76] Punyapornwithaya V Seesupa S Phuykhamsingha S Arjkumpa O Sansamur C Jarassaeng C. Spatio-temporal patterns of lumpy skin disease outbreaks in dairy farms in northeastern Thailand. Front Vet Sci. (2022) 9:957306. doi: 10.3389/fvets.2022.957306 35990277 PMC9386524

[77] Saegerman C Bertagnoli S Meyer G Ganière JP Caufour P De Clercq K . Risk of introduction of lumpy skin disease in France by the import of vectors in animal trucks. PLoS One. (2018) 13:e0198506. doi: 10.1371/journal.pone.0198506, PMID: 29889905 PMC5995388

[78] Saegerman C Bertagnoli S Meyer G Ganière JP Caufour P De Clercq K . Risk of introduction of lumpy skin disease into France through imports of cattle. Transbound Emerg Dis. (2019) 66:957–67. doi: 10.1111/tbed.13111, PMID: 30578746

[79] Sanz-Bernardo B Haga IR Wijesiriwardana N Basu S Larner W Diaz AV . Quantifying and modeling the acquisition and retention of lumpy skin disease virus by Hematophagus insects reveals clinically but not subclinically affected cattle are promoters of viral transmission and key targets for control of disease outbreaks. J Virol. (2021) 95:e02239–20. doi: 10.1128/JVI.02239-20, PMID: 33568514 PMC8104101

[80] Şevik M Doğan M. Epidemiological and molecular studies on lumpy skin disease outbreaks in Turkey during 2014–2015. Transbound Emerg Dis. (2017) 64:1268–79. doi: 10.1111/tbed.12501 27039847

[81] Stojmanovski Z . Space-time permutation model applied to the past outbreak data of lumpy skin disease in the Balkan Peninsula from august 2015 to July 2017. Vet Glas. (2018) 72:44–55. doi: 10.2298/VETGL171027003S

[82] Suparyati S Utami E Muhammad AH. Applying different resampling strategies in random forest algorithm to predict lumpy skin disease. J RESTI Rekayasa Sist Dan Teknol Inf. (2022) 6:555–62. doi: 10.29207/resti.v6i4.4147

[83] Susanti T Susetya H Widayani P Fitria Y Pambudi GT. Risk factors, logistic model, and vulnerability mapping of lumpy skin disease in livestock at the farm level in Indragiri Hulu district, Riau province, Indonesia, in 2022. Vet World. (2023) 16:2071–9. doi: 10.14202/vetworld.2023.2071-2079 38023269 PMC10668545

[84] Swiswa S Masocha M Pfukenyi DM Dhliwayo S Chikerema SM. Long-term changes in the spatial distribution of lumpy skin disease hotspots in Zimbabwe. Trop Anim Health Prod. (2017) 49:195–9. doi: 10.1007/s11250-016-1180-9 27785763

[85] Taylor RA Berriman ADC Gale P Kelly LA Snary EL. A generic framework for spatial quantitative risk assessments of infectious diseases: lumpy skin disease case study. Transbound Emerg Dis. (2019) 66:131–43. doi: 10.1111/tbed.12993, PMID: 30102842

[86] Uddin MA Islam MA Rahman AKMA Rahman MM Khasruzzaman AKM Ward MP . Epidemiological investigation of lumpy skin disease outbreaks in Bangladeshi cattle during 2019–2020. Transbound Emerg Dis. (2022) 69:3397–404. doi: 10.1111/tbed.14696, PMID: 36053488

[87] Wang J Xu Z Wang Z Li Q Liang X Ye S . Isolation, identification and phylogenetic analysis of lumpy skin disease virus strain of outbreak in Guangdong, China. Transbound Emerg Dis. (2022) 69:e2291–301. doi: 10.1111/tbed.14570 35478381

[88] Tuladhar R Singh A Banjara MR Gautam I Dhimal M Varma A . Effect of meteorological factors on the seasonal prevalence of dengue vectors in upland hilly and lowland Terai regions of Nepal. Parasit Vectors. (2019) 12:42. doi: 10.1186/s13071-019-3304-3, PMID: 30658693 PMC6339416

[89] Magombedze G Ferguson NM Ghani AC. A trade-off between dry season survival longevity and wet season high net reproduction can explain the persistence of Anopheles mosquitoes. Parasit Vectors. (2018) 11:576. doi: 10.1186/s13071-018-3158-0, PMID: 30390714 PMC6215619

[90] Wilhelm L Ward MP. The spread of lumpy skin disease virus across Southeast Asia: insights from surveillance. Transbound Emerg Dis. (2023) 2023:1–9. doi: 10.1155/2023/3972359 PMC12016946 40303671

[91] Punyapornwithaya V Arjkumpa O Buamithup N Jainonthee C Salvador R Jampachaisri K. The impact of mass vaccination policy and control measures on lumpy skin disease cases in Thailand: insights from a Bayesian structural time series analysis. Front Vet Sci. (2024) 10:10. doi: 10.3389/fvets.2023.1301546, PMID: 38249552 PMC10797105

[92] Tango T . Spatial scan statistics can be dangerous. Stat Methods Med Res. (2021) 30:75–86. doi: 10.1177/0962280220930562 33595399

[93] Vandenbussche F Mathijs E Philips W Saduakassova M De Leeuw I Sultanov A . Recombinant LSDV strains in Asia: vaccine spillover or natural emergence? Viruses. (2022) 14:1429. doi: 10.3390/v14071429, PMID: 35891412 PMC9318037

[94] Shi M Lin XD Chen X Tian JH Chen LJ Li K . The evolutionary history of vertebrate RNA viruses. Nature. (2018) 556:197–202. doi: 10.1038/s41586-018-0012-7 29618816

[95] Alkhamis MA Li C Torremorell M. Animal disease surveillance in the 21st century: applications and robustness of Phylodynamic methods in recent U.S. human-like H3 swine influenza outbreaks. Front Vet Sci. (2020) 7:176. doi: 10.3389/fvets.2020.00176 32373634 PMC7186338

[96] Stram Y Kuznetzova L Friedgut O Gelman B Yadin H Rubinstein-Guini M. The use of lumpy skin disease virus genome termini for detection and phylogenetic analysis. J Virol Methods. (2008) 151:225–9. doi: 10.1016/j.jviromet.2008.05.003 18582954

[97] Liu P Song Y Colijn C MacPherson A. The impact of sampling bias on viral phylogeographic reconstruction. PLOS Glob Public Health. (2022) 2:e0000577. doi: 10.1371/journal.pgph.0000577 36962555 PMC10021582

[98] Eagles D Deveson T Walker PJ Zalucki MP Durr P. Evaluation of long-distance dispersal of Culicoides midges into northern Australia using a migration model. Med Vet Entomol. (2012) 26:334–40. doi: 10.1111/j.1365-2915.2011.01005.x 22211884

[99] Eagles D Walker PJ Zalucki MP Durr PA. Modelling spatio-temporal patterns of long-distance Culicoides dispersal into northern Australia. Prev Vet Med. (2013) 110:312–22. doi: 10.1016/j.prevetmed.2013.02.022 23642857

[100] Wallinga J Teunis P. Different epidemic curves for severe acute respiratory syndrome reveal similar impacts of control measures. Am J Epidemiol. (2004) 160:509–16. doi: 10.1093/aje/kwh255 15353409 PMC7110200

[101] Pérez-Reche FJ Taylor N McGuigan C Conaglen P Forbes KJ Strachan NJC . Estimated dissemination ratio—a practical alternative to the reproduction number for infectious diseases. Front. Public Health. (2021) 9:9. doi: 10.3389/fpubh.2021.675065, PMID: 34336770 PMC8316631

[102] Lecca LO Paiva MT de Oliveira CSF Morais MHF de Azevedo MI e Bastos C d V . Associated factors and spatial patterns of the epidemic sporotrichosis in a high density human populated area: a cross-sectional study from 2016 to 2018. Prev Vet Med. (2020) 176:104939. doi: 10.1016/j.prevetmed.2020.104939, PMID: 32143029

[103] Cuervo PF Artigas P Lorenzo-Morales J Bargues MD Mas-Coma S. Ecological niche modelling approaches: challenges and applications in vector-borne diseases. Trop Med Infect Dis. (2023) 8:187. doi: 10.3390/tropicalmed8040187, PMID: 37104313 PMC10141209

[104] Elsaid M Nasef MA Huy NT. R0 of COVID-19 and its impact on vaccination coverage: compared with previous outbreaks. Hum Vaccin Immunother. (2021) 17:3850–4. doi: 10.1080/21645515.2020.1865046, PMID: 34612165 PMC8827628

[105] Boonpatcharanon S Heffernan JM Jankowski H. Estimating the basic reproduction number at the beginning of an outbreak. PLoS One. (2022) 17:e0269306. doi: 10.1371/journal.pone.0269306 35714080 PMC9205483

[106] Jewell CP Keeling MJ Roberts GO. Predicting undetected infections during the 2007 foot-and-mouth disease outbreak. J R Soc Interface. (2009) 6:1145–51. doi: 10.1098/rsif.2008.0433, PMID: 19091686 PMC2817150

[107] Firestone SM Hayama Y Bradhurst R Yamamoto T Tsutsui T Stevenson MA. Reconstructing foot-and-mouth disease outbreaks: a methods comparison of transmission network models. Sci Rep. (2019) 9:4809. doi: 10.1038/s41598-019-41103-6 30886211 PMC6423326

[108] Flage R Askeland T. Assumptions in quantitative risk assessments: when explicit and when tacit? Reliab Eng Syst Saf. (2020) 197:106799. doi: 10.1016/j.ress.2020.106799

[109] Lambkin K Hamilton J McGrath G Dando P Draxler R. Foot and mouth disease atmospheric dispersion system. Adv Sci Res. (2019) 16:113–7. doi: 10.5194/asr-16-113-2019

[110] Garner MG Hess GD Yang X. An integrated modelling approach to assess the risk of wind-borne spread of foot-and-mouth disease virus from infected premises. Environ Model Assess. (2006) 11:195–207. doi: 10.1007/s10666-005-9023-5

[111] Zalcman E Hall R Cowled B. Lumpy skin disease risk assessment: a qualitative assessment on unregulated pathways. Canberra, Australia: AusVet Pty Ltd.; (2022). Available at: https://www.agriculture.gov.au/sites/default/files/documents/ausvet-lsd-qualitative-risk-assessment.pdf (accessed July 3, 2024).

[112] Valencia-Rodríguez D Jiménez-Segura L Rogéliz CA Parra JL. Ecological niche modeling as an effective tool to predict the distribution of freshwater organisms: the case of the Sabaleta *Brycon henni* (Eigenmann, 1913). PLoS One. (2021) 16:e0247876. doi: 10.1371/journal.pone.0247876 33657168 PMC7928524

[113] Garner MG Bombarderi N Cozens M Conway ML Wright T Paskin R . Estimating resource requirements to staff a response to a medium to large outbreak of foot and mouth disease in Australia. Transbound Emerg Dis. (2016) 63:e109–21. doi: 10.1111/tbed.12239, PMID: 24894407

[114] Bradhurst RA Roche SE East IJ Kwan P Garner MG. A hybrid modeling approach to simulating foot-and-mouth disease outbreaks in Australian livestock. Front. Environ Sci. (2015) 3:3. doi: 10.3389/fenvs.2015.00017

[115] Feelders A Daniels H Holsheimer M. Methodological and practical aspects of data mining. Inf Manag. (2000) 37:271–81. doi: 10.1016/S0378-7206(99)00051-8

[116] García-Lastra R Leginagoikoa I Plazaola JM Ocabo B Aduriz G Nunes T . Bluetongue virus serotype 1 outbreak in the Basque Country (northern Spain) 2007–2008. Data support a primary vector windborne transport. PLoS One. (2012) 7:e34421. doi: 10.1371/journal.pone.0034421 22479628 PMC3316701

[117] Souley Kouato B De Clercq K Abatih E Dal Pozzo F King DP Thys E . Review of epidemiological risk models for foot-and-mouth disease: implications for prevention strategies with a focus on Africa. PLoS One. (2018) 13:e0208296. doi: 10.1371/journal.pone.0208296, PMID: 30543641 PMC6292601

[118] Hui J Wu Y Zhao F Lei X Sun P Singh SK . Parameter optimization for uncertainty reduction and simulation improvement of hydrological modeling. Remote Sens. (2020) 12:4069. doi: 10.3390/rs12244069

[119] Kieu LM Malleson N Heppenstall A. Dealing with uncertainty in agent-based models for short-term predictions. R Soc Open Sci. (2020) 7:191074. doi: 10.1098/rsos.191074, PMID: 32218939 PMC7029931

[120] Desender K Boldt A Verguts T Donner TH. Confidence predicts speed-accuracy tradeoff for subsequent decisions. Elife. (2019) 8:e43499. doi: 10.7554/eLife.43499, PMID: 31429827 PMC6711665

[121] Geoghegan JL Holmes EC. The phylogenomics of evolving virus virulence. Nat Rev Genet. (2018) 19:756–69. doi: 10.1038/s41576-018-0055-5 30305704 PMC7096893

[122] Saqib SE Yaseen M Visetnoi S Sikandar AS. Epidemiological and economic consequences of lumpy skin disease outbreaks on farm households in Khyber Pakhtunkhwa, Pakistan. Front Vet Sci. (2023) 10:1238771. doi: 10.3389/fvets.2023.1238771, PMID: 38188720 PMC10771306

[123] Uusitalo L Lehikoinen A Helle I Myrberg K. An overview of methods to evaluate uncertainty of deterministic models in decision support. Environ Model Softw. (2015) 63:24–31. doi: 10.1016/j.envsoft.2014.09.017

[124] Mitra ED Hlavacek WS. Parameter estimation and uncertainty quantification for systems biology models. Curr Opin Syst Biol. (2019) 18:9–18. doi: 10.1016/j.coisb.2019.10.006 32719822 PMC7384601

[125] Dubé C Stevenson M Garner M Sanson R Corso B Harvey N . A comparison of predictions made by three simulation models of foot-and-mouth disease. N Z Vet J. (2007) 55:280–8. doi: 10.1080/00480169.2007.36782, PMID: 18059645

[126] Mehta L Srivastava S. Uncertainty in modelling climate change. In: Scoones I Stirling A, editors. The politics of uncertainty: Challenges of transformation. 1st ed. London: Routledge Press (2020). 99–112.

