# Supplementary material for: A systematic review of epidemiological modelling in response to lumpy skin disease outbreaks
Source: Front Vet Sci. 2024 Sep 23;11:1459293. doi: 10.3389/fvets.2024.1459293 (PMC11456570; doi:10.3389/fvets.2024.1459293)
Supplement: Supplementary file 1 [file Presentation_1.pptx]

## Slide 1
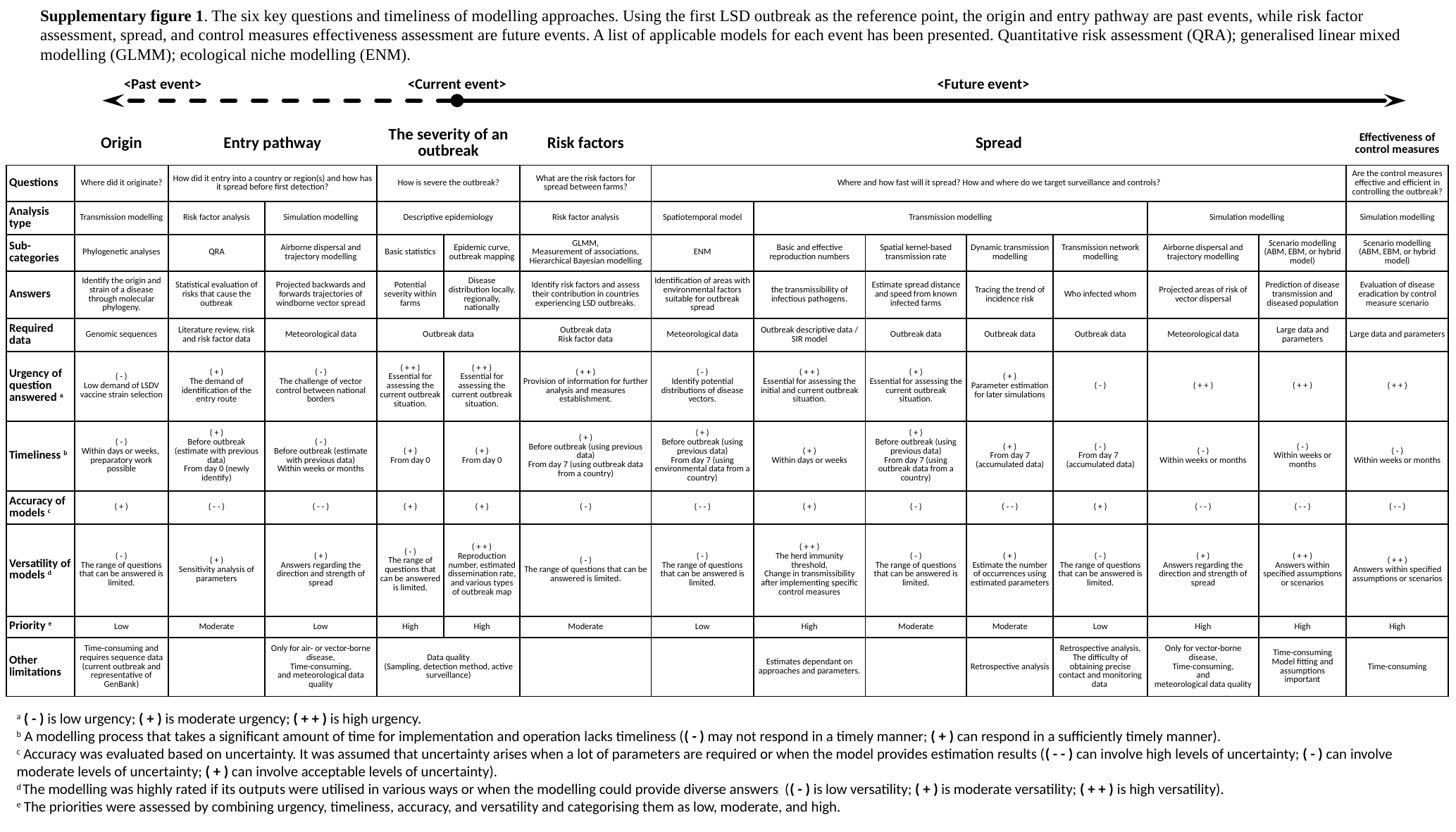

Supplementary figure 1. The six key questions and timeliness of modelling approaches. Using the first LSD outbreak as the reference point, the origin and entry pathway are past events, while risk factor assessment, spread, and control measures effectiveness assessment are future events. A list of applicable models for each event has been presented. Quantitative risk assessment (QRA); generalised linear mixed modelling (GLMM); ecological niche modelling (ENM).
<Current event>
<Future event>
<Past event>
| | Origin | Entry pathway | | The severity of an outbreak | | Risk factors | Spread | Transmission | | | | | | Effectiveness of control measures |
| --- | --- | --- | --- | --- | --- | --- | --- | --- | --- | --- | --- | --- | --- | --- |
| Questions | Where did it originate? | How did it entry into a country or region(s) and how has it spread before first detection? | | How is severe the outbreak? | | What are the risk factors for spread between farms? | Where and how fast will it spread? How and where do we target surveillance and controls? | Where it goes and how fast it goes? | | | | | | Are the control measures effective and efficient in controlling the outbreak? |
| Analysis type | Transmission modelling | Risk factor analysis | Simulation modelling | Descriptive epidemiology | | Risk factor analysis | Spatiotemporal model | Transmission modelling | | | | Simulation modelling | | Simulation modelling |
| Sub-categories | Phylogenetic analyses | QRA | Airborne dispersal and trajectory modelling | Basic statistics | Epidemic curve, outbreak mapping | GLMM, Measurement of associations, Hierarchical Bayesian modelling | ENM | Basic and effective reproduction numbers | Spatial kernel-based transmission rate | Dynamic transmission modelling | Transmission network modelling | Airborne dispersal and trajectory modelling | Scenario modelling (ABM, EBM, or hybrid model) | Scenario modelling (ABM, EBM, or hybrid model) |
| Answers | Identify the origin and strain of a disease through molecular phylogeny. | Statistical evaluation of risks that cause the outbreak | Projected backwards and forwards trajectories of windborne vector spread | Potential severity within farms | Disease distribution locally, regionally, nationally | Identify risk factors and assess their contribution in countries experiencing LSD outbreaks. | Identification of areas with environmental factors suitable for outbreak spread | the transmissibility of infectious pathogens. | Estimate spread distance and speed from known infected farms | Tracing the trend of incidence risk | Who infected whom | Projected areas of risk of vector dispersal | Prediction of disease transmission and diseased population | Evaluation of disease eradication by control measure scenario |
| Required data | Genomic sequences | Literature review, risk and risk factor data | Meteorological data | Outbreak data | Outbreak data | Outbreak data Risk factor data | Meteorological data | Outbreak descriptive data / SIR model | Outbreak data | Outbreak data | Outbreak data | Meteorological data | Large data and parameters | Large data and parameters |
| Urgency of question answered a | ( - ) Low demand of LSDV vaccine strain selection | ( + ) The demand of identification of the entry route | ( - ) The challenge of vector control between national borders | ( + + ) Essential for assessing the current outbreak situation. | ( + + ) Essential for assessing the current outbreak situation. | ( + + ) Provision of information for further analysis and measures establishment. | ( - ) Identify potential distributions of disease vectors. | ( + + ) Essential for assessing the initial and current outbreak situation. | ( + ) Essential for assessing the current outbreak situation. | ( + ) Parameter estimation for later simulations | ( - ) | ( + + ) | ( + + ) | ( + + ) |
| Timeliness b | ( - ) Within days or weeks, preparatory work possible | ( + ) Before outbreak (estimate with previous data) From day 0 (newly identify) | ( - ) Before outbreak (estimate with previous data) Within weeks or months | ( + ) From day 0 | ( + ) From day 0 | ( + ) Before outbreak (using previous data) From day 7 (using outbreak data from a country) | ( + ) Before outbreak (using previous data) From day 7 (using environmental data from a country) | ( + ) Within days or weeks | ( + ) Before outbreak (using previous data) From day 7 (using outbreak data from a country) | ( + ) From day 7 (accumulated data) | ( - ) From day 7 (accumulated data) | ( - ) Within weeks or months | ( - ) Within weeks or months | ( - ) Within weeks or months |
| Accuracy of models c | ( + ) | ( - - ) | ( - - ) | ( + ) | ( + ) | ( - ) | ( - - ) | ( + ) | ( - ) | ( - - ) | ( + ) | ( - - ) | ( - - ) | ( - - ) |
| Versatility of models d | ( - ) The range of questions that can be answered is limited. | ( + ) Sensitivity analysis of parameters | ( + ) Answers regarding the direction and strength of spread | ( - ) The range of questions that can be answered is limited. | ( + + ) Reproduction number, estimated dissemination rate, and various types of outbreak map | ( - ) The range of questions that can be answered is limited. | ( - ) The range of questions that can be answered is limited. | ( + + ) The herd immunity threshold, Change in transmissibility after implementing specific control measures | ( - ) The range of questions that can be answered is limited. | ( + ) Estimate the number of occurrences using estimated parameters | ( - ) The range of questions that can be answered is limited. | ( + ) Answers regarding the direction and strength of spread | ( + + ) Answers within specified assumptions or scenarios | ( + + ) Answers within specified assumptions or scenarios |
| Priority e | Low | Moderate | Low | High | High | Moderate | Low | High | Moderate | Moderate | Low | High | High | High |
| Other limitations | Time-consuming and requires sequence data (current outbreak and representative of GenBank) | | Only for air- or vector-borne disease, Time-consuming, and meteorological data quality | Data quality (Sampling, detection method, active surveillance) | Data quality (Sampling, detection method, active surveillance) | | | Estimates dependant on approaches and parameters. | | Retrospective analysis | Retrospective analysis, The difficulty of obtaining precise contact and monitoring data | Only for vector-borne disease, Time-consuming, and meteorological data quality | Time-consuming Model fitting and assumptions important | Time-consuming |
a ( - ) is low urgency; ( + ) is moderate urgency; ( + + ) is high urgency.
b A modelling process that takes a significant amount of time for implementation and operation lacks timeliness (( - ) may not respond in a timely manner; ( + ) can respond in a sufficiently timely manner).
c Accuracy was evaluated based on uncertainty. It was assumed that uncertainty arises when a lot of parameters are required or when the model provides estimation results (( - - ) can involve high levels of uncertainty; ( - ) can involve moderate levels of uncertainty; ( + ) can involve acceptable levels of uncertainty).
d The modelling was highly rated if its outputs were utilised in various ways or when the modelling could provide diverse answers (( - ) is low versatility; ( + ) is moderate versatility; ( + + ) is high versatility).
e The priorities were assessed by combining urgency, timeliness, accuracy, and versatility and categorising them as low, moderate, and high.
